# Immune checkpoint blockade and CAR-T cell therapy in hematologic malignancies

**DOI:** 10.1186/s13045-019-0746-1

**Published:** 2019-06-11

**Authors:** Hao Wang, Gurbakhash Kaur, Alexander I. Sankin, Fuxiang Chen, Fangxia Guan, Xingxing Zang

**Affiliations:** 1 Department of Microbiology and Immunology, Albert Einstein College of Medicine, Bronx, NY 10461 USA; 2 Department of Medical Oncology, Montefiore Medical Center, Albert Einstein College of Medicine, Bronx, NY 10461 USA; 3 Department of Urology, Montefiore Medical Center, Albert Einstein College of Medicine, Bronx, NY 10461 USA; 4 Ninth People’s Hospital, Shanghai Jiao Tong University School of Medicine, Shanghai, 200011 China; 5 School of Life Sciences, Zhengzhou University, Zhengzhou, 450001 Henan China

**Keywords:** Hematologic malignancies, Immune checkpoints, CAR-T, Immunotherapy, CTLA-4, PD-1, New targets

## Abstract

Harnessing the power of the immune system to recognize and eliminate cancer cells is a longtime exploration. In the past decade, monoclonal antibody (mAb)-based immune checkpoint blockade (ICB) and chimeric antigen receptor T (CAR-T) cell therapy have proven to be safe and effective in hematologic malignancies. Despite the unprecedented success of ICB and CAR-T therapy, only a subset of patients can benefit partially due to immune dysfunction and lack of appropriate targets. Here, we review the preclinical and clinical advances of CTLA-4 and PD-L1/PD-1-based ICB and CD19-specific CAR-T cell therapy in hematologic malignancies. We also discuss the basic research and ongoing clinical trials on emerging immune checkpoints (Galectin-9/Tim-3, CD70/CD27, LAG-3, and LILRBs) and on new targets for CAR-T cell therapy (CD22, CD33, CD123, BCMA, CD38, and CD138) for the treatment of hematologic malignancies.

## Introduction

Our current understanding of hematopoiesis is based on a stem cell model, in which a small pool of multi-potent hematopoietic stem cells (HSCs) self-renew and differentiate into distinct cellular lineages of the blood [1]. This process is tightly regulated to maintain an appropriate number of mature progenies with specific function while not exhausting primitive stem cells [2]. Dysregulation of hematopoiesis results in the development of hematologic malignancy, which is a group of blood cancers arising from cells with reduced capacity to differentiate into mature progeny, leading to the accumulation of immature cells in blood-forming tissues. In 2019, 176,200 new hematologic malignancy cases and 56,770 deaths are projected to occur in the USA according to the data released by the American Cancer Society [3].

Chemotherapy and bone marrow (BM) transplantation are the standard treatments for acute myeloid leukemia (AML), acute lymphoid leukemia (ALL), aggressive Hodgkin’s lymphoma (HL), and Non-Hodgkin’s lymphoma (NHL) such as diffuse large B cell lymphoma (DLBCL) and Burkitt’s lymphoma. Although a temporary remission can be achieved, the risk of relapse remains high because of the existence of chemotherapy-resistant cancer stem cells [4]. Novel methods of immunotherapy, such as immune checkpoint blockade (ICB) and chimeric antigen receptor T (CAR-T) cell therapy have been attracting attention due to their ability to charge the immune system to attack cancer cells.

## Targeting immune checkpoints in hematologic malignancies

**Fig. 1:**
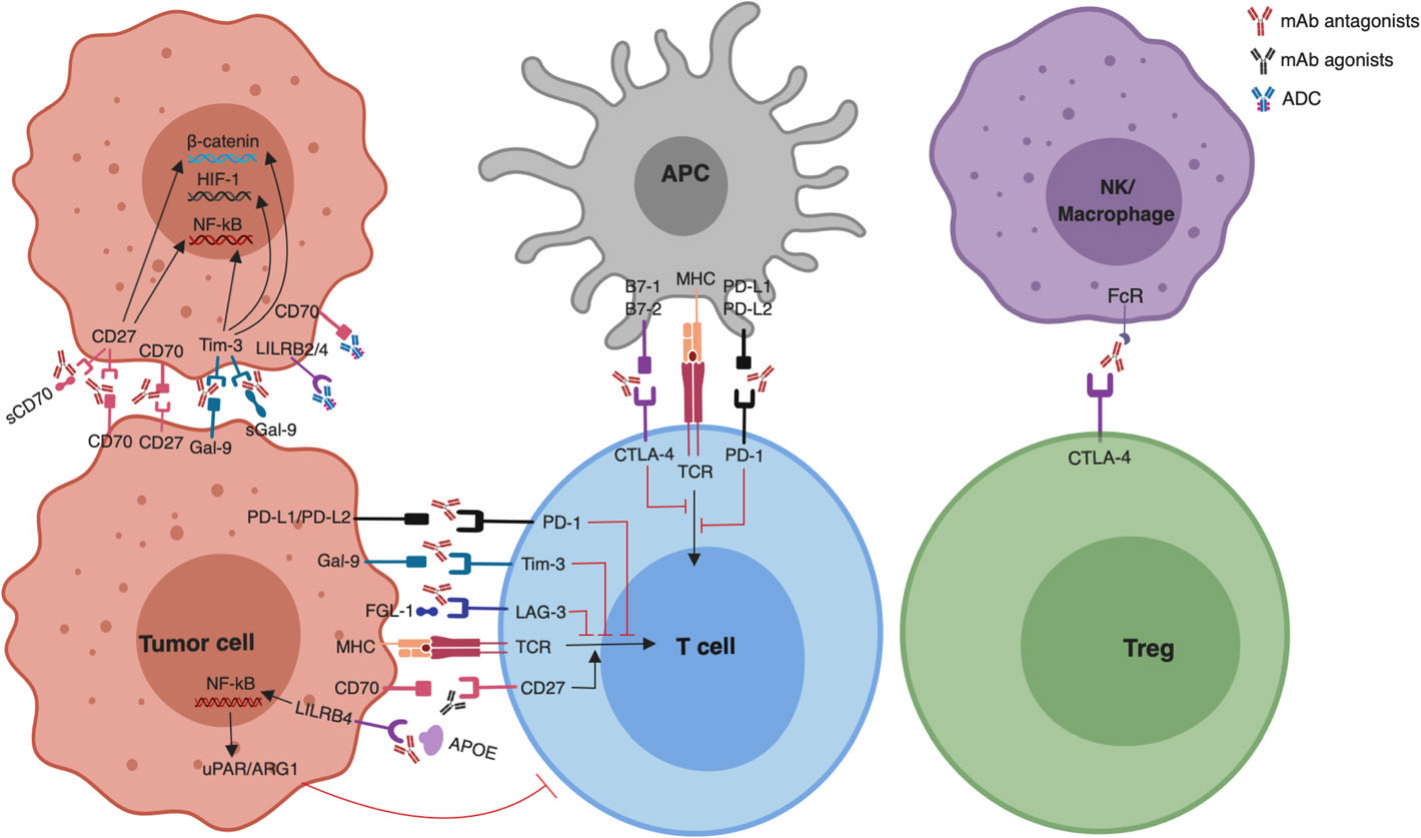
Immune checkpoint blockade (ICB) with mAbs in hematologic malignancies. CD70/CD27 and Galectin-9 (Gal-9)/Tim-3 expression in hematologic malignancies (tumor cell): mAb antagonists inhibit tumor progression by blocking autocrine stimulatory loops, which intrinsically promote tumor cell growth and self-renewal via β-catenin/HIF-1/NF-κB pathways. LILRB2/4 and CD70 expression on tumor cell: antibody-drug conjugate (ADC) specifically binds and kills tumor cells. PD-L1/PD-L2, Gal-9, LILRB4, and MHCII/FGL-1 expression on tumor cell: mAb antagonists targeting their receptors/ligand to neutralize co-inhibitory signals for T cell anti-tumor immune responses. CD27 expression on T cell: mAb agonist promotes T cell response. CTLA-4 expression on T cells: mAb antagonist removes inhibitory T cell signaling and selectively deletes intratumoral regulatory T cells (Treg) via antibody-dependent cell-mediated cytotoxicity (ADCC). sGal-9, soluble Galectin-9; sTim-3, soluble Tim-3; APOE, apolipoprotein E; uPAR, urokinase receptor; ARG1, arginase-1; FGL-1, fibrinogen-like protein 1

T cell activation is a rigorous process regulated by two signals: the T cell receptor (TCR) engaging with peptide/major histocompatibility complex (MHC) results in the first signal; the interaction between CD28 on T cells and its ligand B7-1 (CD80)/B7-2 (CD86) on antigen-presenting cells (APC) stimulates the T cell, serving as the co-stimulatory signal [5]. The B7/CD28 and tumor necrosis factor (TNF) superfamily members are the most extensively studied immune checkpoints over the past two decades. The B7/CD28 family can be divided into three groups based on the phylogenetic analysis [6]. Group I contains B7-1/B7-2/CD28/CTLA-4 and ICOS-L (B7h)/ICOS. Group II includes PD-L1/PD-L2/PD-1. Group III consists of B7H3 (CD276), B7x (B7H4, B7S1), and HHLA2 (B7H5, B7H7)/TMIGD2 (CD28H, IGPR-1). In 1996, James Allison and colleagues first reported that treating tumor-bearing immune competent mice with anti-CTLA-4 antagonist mAb resulted in tumor rejection, suggesting that removing T cell co-inhibitory signal was an effective approach to treat cancer [7]. Subsequent clinical trials based on humanized anti-CTLA-4 mAb (ipilimumab) showed improved overall survival (OS) in patients with metastatic melanoma, thus leading to its approval by the US Food and Drug Administration (FDA) in 2011 [8]. The past 8 years have witnessed the revolutionization of cancer treatment by targeting the immune checkpoint receptors CTLA-4 and PD-1 (nivolumab, pembrolizumab, and cemiplimab), as well as PD-L1 (avelumab, durvalumab, and atezolizumab). Due to their fundamental and translational contributions for identifying and characterizing the function of immune checkpoints in cancer, James Allison and Tasuku Honjo were awarded the 2018 Nobel Prize in Physiology or Medicine [9]. Since ICB mainly relies on the reactivation and expansion of T cells, immunophenotyping of tumor-infiltrating lymphocytes (TILs) during hematologic malignancy progression is therefore of great importance. T cells in both peripheral blood and BM from patients with hematologic malignancies have shown impaired function and abnormal phenotype [10]. These basic and preliminary findings have inspired researchers to evaluate the possibility of ICB in hematologic malignancies following the unprecedented success with ICB in solid tumors (Fig. 1).

### CTLA-4

CTLA-4 is expressed on activated T cells, regulatory T cells (Tregs), and AML blasts [11–13]. Anti-CTLA-4 toxin-conjugated mAb treatment induced dramatic apoptosis in AML cells but was only slightly toxic to normal BM precursors [11]. Furthermore, engagement of CTLA-4 by its specific ligands B7-1 and B7-2 induced apoptosis in patient-derived AML cells via a T cell-independent pathway [12]. On the other hand, in murine C1498 myelogenous leukemia model, B7-1^+^ C1498 cells grew progressively; B7-2^+^ C1498 cells, however, were rejected spontaneously through a CD8^+^ T cell-mediated killing. By using anti-CTLA-4 mAb to specifically block the B7-1/CTLA-4 interaction, a significantly higher rate of rejection of B7-1^+^ C1498 tumor was observed, indicating that B7-1 delivered negative signal to T cell immunity via CTLA-4 [14]. Another group found that in murine DA1-3b AML model, B7-1 and PD-L1 expression were increased in leukemic cells, which were more resistant to host immune responses and thus resulting in worse survival. Blocking PD-L1, B7-1, or CTLA-4 enhanced cytotoxic T cell-mediated lysis and prolonged survival of DA1-3b AML mice [15]. AML patients with the CTLA-4 CT60 AA genotype had increased risk of leukemic relapse after standard chemotherapy and lower overall survival at 3 years. The CTLA-4 CT60 AA genotype has been described to produce a more soluble form of CTLA-4, which is able to suppress proliferation of autoreactive T cells [16].

In HL, TILs were enriched for CTLA-4^+^ Tregs [17]. T cells from patients with chronic lymphocytic leukemia (CLL) had abnormal upregulation of CTLA-4, which was positively correlated with an increased portion of Tregs and advanced Rai stage [18]. Co-culture of primary T cells with CLL-derived CTLA-4^+^ Mec1 cells resulted in reduced production of interleukin-2 (IL-2), suggesting that leukemic cells expressing CTLA-4 inhibited T cell co-stimulation [19]. Furthermore, polymorphisms of CTLA-4 were found to be associated with NHL [20]. CTLA-4 has also been reported to upregulate in multiple myeloma (MM) patients [21].

### PD-L1/PD-L2/PD-1

#### MDS/AML

PD-L1 expression in murine leukemia cell line C1498 was upregulated in vivo, and blocking PD-L1/PD-1 pathway resulted in decreased AML burden and longer survival time [22]. In myelodysplastic syndromes (MDS) and AML patient samples, PD-L1 was detectable (> 2% PD-L1^+^ cells) in 100% of patients with common expression on non-tumor hematopoietic cells, while PD-L2 expression was largely absent [23]. PD-L1 expression on AML cells is significantly higher in the relapse setting than at the newly diagnosed stage [24, 25]. In BM aspirates from patients with TP53 mutation, PD-L1 positivity was more frequently noted [25]. Higher PD-L1 expression level was positively correlated with poor risk cytogenetic and molecular abnormalities [25, 26]. In a similar manner to solid tumor, interferon-γ (IFN-γ) induced PD-L1 expression on AML cells protected them from cytotoxic T cell lysis [27]. In BM aspirates from AML patients, T cell subsets, such as CD4^+^ effector T cells, CD8^+^ T cells, and Tregs, had significantly higher PD-1 expression in untreated and relapsed AML patients compared with healthy donors [28]. PD-1 expression on CD4^+^ and CD8^+^ T cells was upregulated at relapse after allogeneic stem cell transplantation (allo-SCT) [29]. In peripheral blood of patients with chronic myeloid leukemia (CML), PD-1 expression on CD8^+^ T cells was higher in comparison with healthy donors. In CML mouse model, PD-1 was highly expressed on CML-specific cytotoxic T cells, while PD-L1 expression was higher in blast crisis CML (bcCML) than chronic phase CML (cpCML), indicating that CML cells utilized PD-L1 to avoid immune surveillance. PD-1-deficient mice with bcCML survived significantly longer than wild type mice, suggesting that myeloid leukemia cells impaired host immune responses via PD-L1/PD-1 pathway [30]. Hence, the upregulation of PD-L1 on MDS/AML cells leads to immune escape and supports the potential benefit of using PDL-1/PD-1 inhibitors to treat MDS/AML.

Single agent nivolumab (humanized anti-PD-1 IgG4 mAb) as maintenance therapy demonstrated a complete remission (CR) rate of 71% in 14 transplant ineligible patients with high-risk features including adverse cytogenetics, treatment-related AML, and history of prior relapse (Table 1) [33]. Early results of pembrolizumab (humanized anti-PD-1 IgG4 mAb) plus cytarabine yielded CR rate of 35% and minimal residual disease (MRD)-negative remission in 56% of patients (Table 1) [34]. Idarubicin plus cytarabine and nivolumab in newly diagnosed AML reported complete remission or complete remission with incomplete count recovery (CR/CRi) in 34 of 42 patients and MRD-negative remission in 18 patients. Furthermore, the median relapse-free survival for the complete responders was 18.5 months. The risk of graft versus host disease (GvHD) was not significantly elevated in the 18 patients who proceeded to allo-SCT. Interestingly, baseline BM analysis of those who achieved CR/CRi had a higher frequency of CD3^+^ T cell infiltrate as compared to non-responders who had higher number of CD4^+^ effector T cells co-expressing markers of an exhausted phenotype (Table 1) [35, 36]. While the use of nivolumab and ipilimumab (humanized anti-CTLA-4 IgG1 mAb) in the post allo-SCT relapse setting in hematologic malignancies has demonstrated potent anti-tumor effects, significant immune-related adverse events (irAE) have also been reported (Table 1) [31, 49, 50]. Ipilimumab use with various hematologic malignancies resulted in significant irAE including 1 death, GvHD leading to drug discontinuation in 4 patients, pneumonitis and colitis [31]. In addition, fatal acute respiratory distress syndrome (ARDS), antiphospholipid syndrome, fatal acute grade 3 GvHD, and worsening of chronic GvHD were reported with nivolumab use in two other clinical trials [49, 50]. These data highlight a need for caution of T cell-mediated GvHD when treating patients with ICB after allo-SCT. Mechanistically, one study has demonstrated that persistent expression of PD-L1 by parenchymal cells reduces the proliferation of donor-derived CD8^+^ T cells in GvHD target tissues, leading to amelioration of GvHD in a mouse model [51]. Another group has showed similar result that elevated levels of PD-L1 from organ-specific microenvironments (e.g., lymph nodes) dampen cytotoxic T lymphocyte (CTL)-mediated GvHD after allo-SCT [52]. Reduced CTL activity in lymph nodes, however, also contributed to local tumor escape, which could be reversed by anti-PD-1 blockade [52]. It would be important to balance the possible risk of exacerbating GvHD and achieving maximum tumor killing.

**Table 1:** CTLA-4 and PD-1 inhibitors in hematologic malignancies

Trial	Phase	*n*	Disease	Patient characteristics	Intervention	Response	Ref.
NCT01822509	I	28	Post allo-SCT relapsed HM	AML (16), leukemia cutis (3), myeloid sarcoma (1), HL (7), NHL (4), MDS (2), MM, MPN, ALL (1 each)	Ipilimumab	ORR/CR/PR/SD: 32%/23%/9%/27%	[31]
NCT02397720	II	70	RR AML	2°AML (44%), adverse risk CG (34%), TP53 (16 patients)	Nivolumab + azacytidine	ORR/CR/PR/HI/SD: 33%/22%/1.4%/10%/8.5%	[32]
		14	RR AML in 1st or 2nd relapse	2°AML (36%), adverse CG (25%), TP53 (5 patients)	Nivolumab + ipilimumab + azacytidine	ORR/CR/SD: 43%/43%/14%	[32]
NCT02532231	II	14	High-risk AML in CR, ineligible for SCT	Adverse CG (29%), TP53 (3 patients)	Nivolumab maintenance	CRd: 71% at 12 months 12 and 18 months OS: 86% and 67%, respectively	[33]
NCT02768792	II	26	RR AML	2°AML (38%), adverse CG (46%)	Pembrolizumab + cytarabine	ORR/CR/PR: 42%/35%/45% MRD: 5/9 patients in CR	[34]
NCT02464657	I/II	44	Newly diagnosed AML and MDS	De novo AML (32), 2°AML (7), t-AML (3), high-risk MDS (2) Adverse CG (29%), TP53 (8 patients)	Idarubicin, cytarabine + nivolumab	CR/CRi: 77% MRD negative: 18/34 patients in CR	[35, 36]
CheckMate-039	I	23	RR HL	Nodular sclerosing (22), mixed cellularity (1), 78% relapse after SCT/BV	Nivolumab	ORR/CR/PR/SD: 87%/17%/70%/13% 86% PFS rate at 24 weeks 7/20 responses lasted > 1.5 years	[37, 38]
		81	RR NHL	FL (10), DLBCL (11), MF (13), PTCL (5), MM (27), other B-NHL (10), T-NHL (5)	Nivolumab	ORR/CR/PR/SD FL: 40%/10%/30%/60% DLBCL: 36%/18%/18%/27% MF: 15%/0%/15%/69% PTCL: 40%/0/40%/0 MM: 4%/0/0/63% Other B-NHLs: 0/0/0/70% T NHL: 0/0/0/20%	[39]
		65	RR NHL	HL (31), MM (17), PMBCL (1), B-NHL (15), T-NHL (11)	Nivolumab + ipilimumab	ORR/CR/PR/SD HL: 74%/19%/55%/10% MM: 0/0/0/1 B- NHL: 20%/0/20%/7% T-NHL: 9%/0/9%/36%	[40]
CheckMate-205		243	RR HL	Cohort 1: brentuximab vedotin naïve (63) Cohort 2: brentuximab vedotin after auto-SCT (80) Cohort 3: brentuximab vedotin before and or after auto-SCT (100)	Nivolumab	ORR/CR/PR/SD Overall: 69%/16%/53%/19% Cohort 1: 65%/29%/37%/24% Cohort 2: 68%/13%/55%/21% Cohort 3: 73%/12%/61%/15%	[41, 42]
KEYNOTE-13	I	31	RR HL	Post brentuximab vedotin relapse (100%) Post auto-SCT (71%)	Pembrolizumab	ORR/CR/PR/SD: 65%/16%/48%/23%	[43]
		21	RR PBMCL	SCT ineligible (62%)	Pembrolizumab	ORR/CR: 48%/33%	[44]
KEYNOTE-087	II	210	RR HL	Cohort 1: auto-SCT and brentuximab vedotin (69) Cohort 2: salvage CT + brentuximab vedotin (81) Cohort 3: auto-SCT only (60)	Pembrolizumab	ORR/CR/PR: 72%/28%/44% ORR/CR: Cohort 1: 77%/26% Cohort 2: 67%/26% Cohort 3: 73%/32%	[45, 46]
KEYNOTE-170	II	53	RR PBMCL	Auto-SCT ineligible (74%)	Pembrolizumab	ORR/CR: 45%/13%	[44]
NCT02572167	I/II	62	RR HL		Pembrolizumab + brentuximab vedotin	ORR/CR/PR/SD: 82%/61%/21%/8%	[47]
ECOG-ACRIN E4412	I	22	RR HL		Nivolumab + ipilimumab + brentuximab vedotin	ORR/CR: 82%/68%	[48]

*Abbreviations*: *CT* chemotherapy, *HL* Hodgkin’s lymphoma, *FL* follicular lymphoma, *DLBCL* diffuse large B cell lymphoma, *PTCL* peripheral T cell lymphoma, *CTCL* cutaneous T cell lymphoma, *MM* multiple myeloma, *PMBCL* primary mediastinal B cell lymphoma, *AML* acute myeloid leukemia, *MDS* myelodysplastic syndrome, *HM* hematologic malignancies, *MF* mycosis fungoides, *NHL* non-Hodgkin’s lymphoma, *RR* relapsed refractory, *auto-SCT* autologous stem cell transplantation, *CG* cytogenetics, *CRd* complete response duration, *CR* complete remission, *CRi* complete remission with insufficient count recovery, *PR* partial response, *SD* stable disease, *HI* hematologic independence

#### Hodgkin’s lymphoma

PD-L1/PD-L2 expression is increased on HL cell lines and malignant Reed Sternberg (RS) in classical HL (cHL), due to upregulation and amplification of 9p24.1 JAK and MEK/ERK signaling [53, 54]. Although cHL does not have a high mutational burden, a necessary biomarker predicting responses to ICB, high frequency of PD-L1/PD-L2/PD-1/JAK2 genetic alterations in RS cells and high proportion of PD-1^+^ TILs determine sensitivity to PD-L1/PD-1 inhibitors [55, 56]. Receptor PD-1 was markedly increased on TILs as well as peripheral T cells of HL patients [55, 57]. Functionally, mAb targeting PD-L1 was able to inhibit tyrosine phosphorylation of SHP-2 and restore the production of IFN-γ by tumor-infiltrating T cells [57]. Within the tumor microenvironment (TME) of cHL, PD-1 and PD-L1 were elevated on natural killer (NK) cells and tumor-associated macrophages (TAMs), respectively. As expected, PD-1 inhibition reactivated both T and NK cells by blocking interactions between PD-1^+^ T/NK cells and PD-[39]L1^+^ malignant B cells/TAMs [58]. In addition, expanded numbers of CD4^+^PD-1^−^ Th1-polarized Tregs and PD-1^+^ differentiated T effectors were observed within the TME of cHL, where these cells might utilize PD-L1/PD-1 pathway to exert complementary mechanisms to suppress host anti-tumor immune responses [59].

Clinically, both pembrolizumab and nivolumab showed favorable responses and acceptable safety profile in patients with cHL that has relapsed or progressed after autologous stem cell transplantation (auto-SCT) and brentuximab vedotin (BV), leading to their approval in 2016 by US FDA. The phase I clinical trials, KEYNOTE-013 with pembrolizumab and CheckMate 039 with nivolumab, produced overall response rates (ORRs) of 65% (CR 21%) and 87% (CR 17%) in relapsed and refractory (RR) HL, respectively (Table 1) [37, 38, 43]. CheckMate-205, the phase II multi-cohort study of 243 patients with BV naïve-cohort A, BV after auto-SCT cohort B, and BV before and after auto-SCT cohort C, demonstrated ORR of 69% and a median duration of response (DOR) of 16.6 months (Table 1) [41]. Correlative studies of 45 available tumor samples showed concordant alteration of the PD-L1 and PD-L2 loci in the RS cells. Fluorescence in situ hybridization of the RS cells showed 26 cases with copy gain of PD-L1/PD-L2, 12 cases with PD-L1/PD-L2 amplification, and 7 cases with polysomy 9. Furthermore, complete responders had higher PD-L1 than non-responders [42]. Similarly, KEYNOTE-087, the multi-cohort phase II trial with pembrolizumab monotherapy in RR HL patients who progressed after auto-SCT and subsequent BV therapy (cohort 1), salvage chemotherapy and BV (cohort 2), or auto-SCT but no BV (cohort 3), demonstrated ORR of 72% and CR rate of 28% with a median DOR of 11.1 months (Table 1) [45, 46]. Combination therapy of ipilimumab plus nivolumab has also shown efficacy with ORR of 74% in HL (CheckMate 039, Table 1) [40].

Nivolumab plus BV produced ORR of 82% and CR rate of 61% as the first-line salvage therapy (Table 1) [47]. ECOG-ACRIN E4412 study of nivolumab, ipilimumab, and BV demonstrated ORR of 82% (18/22), with a CR rate of 68% (15/22) (Table 1) [48]. Nivolumab followed by treatment with adriamycin, bleomycin, vinblastine, and dacarbazine (ABVD) for patients at high risk of relapse (NCT03033914) and pembrolizumab for patients unsuitable for ABVD (PLIMATH NCT03331731) are being explored in the first-line setting for HL. Pembrolizumab (NCT02684292) and nivolumab (CheckMate-812 NCT03138499) with or without BV are being evaluated in phase III clinical trials in the relapsed setting as well (Table 1).

#### Non-Hodgkin’s lymphoma

In contrast to HL, PD-L1 expression in NHL is markedly heterogeneous. Of two distinct clinical subtypes of DLBCL, PD-L1 expression was rarely detected in germinal center B cell-like (GCB) subtype, while 57% of activated B cell-like DLBCL samples were PD-L1 positive [60]. Other studies showed similar low expression of surface PD-L1 and soluble PD-L1, and the surface PD-L1 expression was positively associated with the number of PD-1^+^ TILs and inversely correlated with the number of Tregs in GCB-DLBCL [61, 62]. In a small number of follicular lymphoma (FL) patients, PD-L1 expression was high [63]. PD-1 expression on TILs of FL was abundant but with complicated expression patterns: many cell types, including CD4^+^ Th1 cells, CD8^+^ cytotoxic T cells, and Tregs, expressed PD-1 [64]. In CLL, histiocytes, not tumor cells, were the main source of PD-L1 expression within the TME [65]. Low numbers of PD-1^+^ TILs were observed, which had controversial association results among different contexts [56]. One study has shown that PD-1 expression was increased on CD4^+^ and CD8^+^ T cells, and the crosstalk between PD-L1 on CLL and PD-1 on CD8^+^ T cells resulted in decreased IFN-γ production [66].

Although PD-L1 expression is heterogeneous among MM patients, many studies have shown that PD-L1 expression is limited to malignant plasma cells (PCs), and PD-L1 overexpression is associated with increased risk of progression from smoldering multiple myeloma to MM [62, 67]. However, other groups detected very low PD-L1 expression on normal PCs and did not observe significant upregulation of PD-L1 on malignant PCs from MM patients, which could explain why nivolumab monotherapy and in combination with ipilimumab had no significant therapeutic activity in a phase I CheckMate-039 study treating RR MM patients [68]. PD-1 was upregulated on CD4^+^ T cells, CD8^+^ T cells, and NK cells within the BM of MM [68–70]. PD-1^+^ NK and T cells were less proliferative and cytotoxic, which could be reversed by anti-PD-L1/PD-1 blocking in vitro and in vivo [68, 70]. Furthermore, lenalidomide, an immunomodulatory drug (IMiD), reduced expression of PD-1 on T and NK cells and downregulated PD-L1 expression on PCs and myeloid-derived suppressive cells (MDSCs) [67, 69, 71]. As a result, combined blockade of PD-L1/PD-1 and lenalidomide enhanced granzyme B and IFN-γ production by T and NK cells and inhibited MDSC-mediated MM progression [67, 71].

Pembrolizumab is approved for RR primary mediastinal B cell lymphoma (PMBCL) based on ORRs of 48% (CR 31%) in KEYNOTE-13 and ORRs of 45% in phase II, KEYNOTE-170 studies (Table 1) [44]. CheckMate-039 also evaluated the efficacy of single agent nivolumab in NHL and demonstrated ORRs of 40% in FL, 36% in DLBCL, 15% in mycosis fungoides, and 40% in peripheral T cell lymphoma (PTCL) (Table 1) [39]. Furthermore, the nivolumab plus ipilimumab cohort of CheckMate-039 reported ORRs of 20% in FL/DLBCL and 9% in transplant-naïve T-NHL patients in 65 patients who had a median number of 4 prior therapies [40]. Nivolumab monotherapy in patients with RR DLBCL who were ineligible for auto-SCT and those with post auto-SCT relapse produced ORRs of 3% and 10%, respectively. Furthermore, median progression-free survival (PFS) and OS were 1.9 and 12.2 months in the post auto-SCT relapse cohort and 1.4 months and 5.8 months in the auto-SCT ineligible group, respectively [72]. Similarly, pembrolizumab maintenance in post auto-SCT chemosensitive patients failed to meet its primary end point as well [73]. In RR FL, pembrolizumab plus rituximab (chimeric anti-CD20 IgG1 mAb) showed ORR of 67% (CR 50%, PR 17%) in patients and a median PFS of 11.4 months. Interestingly, PDL-1 expression was not associated with response [74]. Nivolumab plus ibrutinib demonstrated responses 61% in patients with high-risk CLL/small lymphocytic leukemia (SLL), 33% with FL, 36% with DLBCL, and 65% of patients with Richter’s transformation [75]

Unlike single agent PD-1 blockade that produced minimal responses in RR MM, the combination of PD-1 inhibition with IMiDs was efficacious and produced ORRs of 50–60% [76]. Pembrolizumab monotherapy in patients who did not achieve CR prior to transplant produced a CR rate of 31% and MRD-negative rate of 41% [77]. In addition, pembrolizumab plus lenalidomide and dexamethasone in high-risk patients within 3–6 months of auto-SCT led to stringent CR in 33% patients and 4 patients achieving MRD-negative remission [78]. Despite the obvious preclinical anti-tumor effects of PD-1 blockade and positive results from earlier trials in MM, phase III clinical trials involving combination therapy of PD-1 blockade with IMiDs were placed on hold by the FDA in 2017 due to increased rate of adverse effects observed in KEYNOTE-183 (NCT02576977) and KEYNOTE-185 (NCT02579863) [79]. The pembrolizumab arm in KEYNOTE-183 (pembrolizumab plus pomalidomide and low-dose dexamethasone) experienced myocarditis, hepatitis, Steven Johnson syndrome, hyperthyroidism, pneumonitis, and 2 treatment-related deaths [79]. KEYNOTE-185 (lenalidomide plus pembrolizumab plus low-dose dexamethasone) reported 6 treatment-related deaths, with 4 being due to either cardiac arrest (1), pneumonia (1), myocarditis (1), and cardiac failure [79].

Several ongoing trials are assessing the combination of PD-1 or PD-L1 inhibition with conventional chemotherapy in untreated DLBCL (NCT 03003520) or as consolidation therapy in NHL (NCT03620578) (Table 2). The JAVELIN study (NCT 02951156) is a phase Ib trial assessing efficacy and safety of immunotherapy-based regimens containing avelumab (human anti-PD-L1 IgG1 antibody) in combination with utomilumab (4-1BB agonist), azacytidine (AZA), rituximab, and/or conventional chemotherapy in patients with RR DLBCL (Table 2) [80].

**Table 2:** Selected ongoing clinical trials targeting immune checkpoints

Clinical trial	Phase	*n*	Disease	Intervention	Sponsor/collaborators
NCT03630159	I	32	DLBCL	Tisagenlecleucel + pembrolizumab	Novartis
NCT03620578	II	102	NHL, HGBCL	DA-R-EPOCH induction followed by nivolumab consolidation	Stichting Hemato Oncologie voor Volwassenen Nederland
NCT03121677	I	20	FL	Nivolumab/poly-ICLC/vaccine/±rituximab	Washington University Bristol Myers Squibb
NCT03046953	II	35	RR T cell lymphoma	Avelumab	University of Birmingham Bloodwise/ Pfizer/UK
NCT03003520	II	46	Untreated high-risk DLBCL	Durvalumab + R-CHOP or durvalumab + R-CHOP + lenalidomide	Celgene
NCT02935361	I/II	72	CMML, MDS, relapsed AML	Guadecitabine and atezolizumab	USC/NCI/Van Andel Research Institute
NCT02733042	I/II	106	Lymphoma/CLL	Durvalumab as monotherapy, durvalumab + ibrutinib Durvalumab + bendamustine ± rituximab Durvalumab and lenalidomide ± rituximab	Celgene
NCT02684292	III	300	RR HL	Pembrolizumab vs brentuximab vedotin	Merck Sharp & Dohme Corp.
NCT02603419	I	33	RR HL	Avelumab	Pfizer
NCT01896999	I/II	189	RR HL	Brentuximab vedotin and nivolumab ± ipilimumab	NCI
NCT02951156	III	28	RR DLBCL	Phase I: avelumab/utomilumab/rituximab vs avelumab/utomilumab/azacytidine vs avelumab/rituximab/bendamustine Phase III: any of the above combinations vs. rituximab/bendamustine or rituximab/gemcitabine/oxaliplatin	Pfizer/EMD Serono
NCT01592370	I/II	375	NHL/HL/MM	Nivolumab monotherapy, nivolumab + lirilumab, nivolumab + ipilimumab, daratumumab vs nivolumab + daratumumab, nivolumab + daratumumab + pomalidomide + dexamethasone vs. nivolumab daratumumab	Bristol Myers Squibb/Janssen
NCT03390296	II	138	RR AML	Arm A: PF-04518600 Arm B: PF-04518600 + avelumab Arm C: PF-04518600 + azacytidine Arm D: PF-04518600 + utomilumab Arm E: avelumab + utomilumab Arm F: PF-04518600 + azacytidine + avelumab Arm G: gemtuzumab ozogamicin + glasdegib Arm H: glasdegib + avelumab	M.D. Anderson Cancer Center/Pfizer
LAG-3 and TIM3					
NCT03489369	I	30	Metastatic solid tumor and lymphoma	Sym022 (anti-LAG-3)	Symphogen
NCT03489343	I	48	Metastatic solid tumor and lymphoma	Sym023 (anti-TIM3)	Symphogen
NCT03311412	I	102	Metastatic solid tumor and lymphoma	Sym021 (anti-PD-1) monotherapy or in combination with Sym022 (anti-LAG3) or Sym023 (anti-TIM3)	Symphogen
NCT02061761	I/II	132	Hematologic malignancies	BMS 986016 (anti-LAG3) ± nivolumab (BMS-936558)	Symphogen
NCT03005782	I	546	Malignancies	REGN3767 (anti-LAG-3 Ab ± REGN2810 (anti-PD-1)	Regeneron Pharmaceuticals/Sanofi
NCT03219268	I	243	Solid and hematologic malignancies	MGD013 DART (PD-1 and LAG-3 antibody)	MacroGenics
CD27					
NCT03307746	I/II	40	B cell lymphoma	Varlilumab plus rituximab	Celldex Therapeutics/National Health Service Trust-UK
NCT01460134	I	90	CD27^+^ B and T cell lymphoma, Burkitt’s lymphoma, solid malignancies, CNS lymphoma	Varlilumab	Celldex Therapeutics
NCT03038672	II	106	RR aggressive B cell lymphomas	Varlilumab plus nivolumab	NCI
CD70					
NCT03030612	I/II	36	AML and high-risk MDS	ARGX-110 plus azacytidine	Argenx BVBA
NCT01813539	I/II	100	Advanced cancers	ARGX-110	Argenx BVBA

*Abbreviations*: *HGBCL* high-grade B cell lymphoma (c-myc, bcl2^+^); *DLBCL* diffuse large B cell lymphoma; *AML* acute myeloid leukemia; *MDS* myelodysplastic syndrome; *HL* Hodgkin’s lymphoma; *CMML* chronic myelomonocytic leukemia; *MM* multiple myeloma; *NHL* non-Hodgkin’s lymphoma; *CLL* chronic lymphocytic leukemia; *LAG-3* lymphocyte-activation gene 3; *DA-R-EPOCH* dose-adjusted rituximab, etoposide, prednisone, vincristine, cyclophosphamide, doxorubicin; *R-CHOP* rituximab, cyclophosphamide, doxorubicin, vincristine, prednisone

### Galectin-9/Tim-3

T cell immunoglobulin and mucin-domain containing-3 (Tim-3) shares a similar expression pattern as PD-1 on T cells within the TME, where it functions as a co-inhibitory receptor, thus inhibiting T cell proliferation and cytokine production [81]. Galectin-9, one of the ligands of Tim-3, negatively regulates T cell immunity [82]. PD-1^high^Tim-3^+^ T cell subsets were functionally deficient and were strongly associated with leukemia relapse in AML patients after allo-SCT [83]. The frequency of PD-1^+^Tim-3^+^ T cell subsets, including CD8^+^ T cells, CD4^+^ effector T cells, and Tregs, was increased in relapsed and new AML in comparison with healthy donors [25]. Interestingly, surface expression of Tim-3 was significantly elevated in CD34^+^CD38^−^ AML leukemia stem cells (LSCs) and CD34^+^CD38^+^ leukemic progenitors, but not in CD34^+^CD38^−^ normal HSCs or most portion of CD34^+^CD38^+^ normal progenitors [84]. Another report showed increased levels of soluble Galectin-9 and Tim-3 in the plasma of AML patients compared with healthy donors [85]. Xenograft experiments demonstrated that Tim-3^+^ AML cells were able to initiate human AML in NSG mice and anti-Tim-3 mAb treatment dramatically depleted LSCs and leukemic burden in primary and secondary NSG recipients [84]. Of note, it is inferred that Galectin-9/Tim-3 pathway enhances AML progression via both immune-cell-dependent and immune-cell-independent manners: AML cells take advantage of self-secreted Galectin-9/Tim-3 to attenuate cytotoxic activity of T cells and NK cells; some pathways such as NF-κB, β-catenin, PI3 Kinase/mTOR, and HIF-1 pathways are intrinsically activated with the ligation of Tim-3 by soluble Galectin-9 in human AML cells. As a result, Galectin-9/Tim-3 autocrine loop promotes self-renewal of LSCs [86, 87]. Given that LSCs were considered to be responsible for the relapse of AML after standard therapies, targeting Galectin-9/Tim-3 pathway represents a promising approach in eliminating LSCs. In terms of other hematologic malignancies, Tim-3 was not only detected on tumor cells in DLBCL and HL, but also observed on TILs where it served as a T cell exhaustion marker [88, 89].

Sym023 (NCT03489343), an anti-Tim-3 mAb as single agent or in combination with Sym021, a PD-1 antibody, (NCT03311412) is in phase I clinical trials for both solid and hematologic malignancies (Table 2).

### CD70/CD27

CD27 (also known as TNFRSF7), one of the TNF receptor family members, works as a positive regulator of T cell immunity by CD70 (TNFSF7) engagement [90]. CD27 is constitutively expressed on naïve T cells as well as HSCs. CD27 remains expressed on stem-like memory cells and central-memory-like cells, whereas it is downregulated on effector cells [91]. With regard to hematopoiesis, the CD70/CD27 interaction negatively mediates leukocyte differentiation and decreases myeloid colony-forming capacity of BM progenitor cells [92]. Besides its functions in modulating normal HSC self-renewal and differentiation, CD70/CD27 signaling also promotes LSC growth and disease progression in murine model and leukemia patients [93–95]. In a BCR/ABL-induced CML-like disease murine model, CD27 was expressed by LSCs (defined as Lin^−^Sac-1^−^c-Kit^high^) and leukemia progenitors, where CD27 signaling enhanced proliferation and cell cycle progression in a Wnt/β-catenin-dependent manner [93]. Furthermore, CD70 was induced in LSCs by upregulating transcription factor specificity protein 1 in tyrosine kinase inhibitor-treated CML patients, triggering CD27 signaling which compensated Wnt pathway and therefore ultimately causing relapse [94]. Combining anti-CD70 mAb blockade with imatinib therapy effectively promoted cell death of human CD34^+^ CML stem/progenitor cells in vitro, as well as in a patient-derived xenograft model [94]. More recently, both AML stem/progenitor cells and blasts were found to express CD70 and CD27, while normal HSCs and progenitor cells were negative. In primary AML patient samples, CD70/CD27 signaling enhanced symmetric cell divisions and proliferation by activating canonical Wnt pathway via TRAF2 and TNIK [95]. In addition, mAbs against either CD70 or CD27 have been evaluated in hematological malignancies. For example, a human anti-CD27 mAb eliminated CD27-expressing lymphoma and leukemia via multiple mechanisms: antibody-dependent cellular cytotoxicity (ADCC) and enhancing co-stimulation of T cells [96]. Both anti-CD70 mAb and anti-CD70 antibody-drug conjugates (ADCs) have shown significant anti-tumor effects in xenograft models [97]. In B cell NHL, preexisting and TGF-β-induced intratumoral CD70^+^ effector memory T cells show exhausted phenotype, expressing high levels of PD-1 and Tim-3 [98]. Interestingly, CD27 on malignant B cells triggers CD70 reverse signaling in NK cells, resulting in increased numbers of tumor-infiltrating activated NK cells and prolonged survival of CD27-expressing lymphoma-bearing mice [99].

Based on preclinical data, anti-CD70 therapy is being studied in AML/MDS and T cell lymphomas. ARGX-110, which blocks CD27/CD70 signaling, demonstrated ORR of 23% in heavily pre-treated patients with CD70 expressing advanced cutaneous T cell lymphoma of different subtypes and stages in a phase I/II clinical trial [100]. A phase II clinical trial (NCT03030612) of ARGX-110 with AZA in AML/MDS is also underway. ADCs of CD70 mAb with a small molecule, MED-2460 (MDX-1203; NCT00944905), with pyrrolobenzodiazepine (SGN-70A, NCT02216890) and monomethyl auristatin (SGN-75, NCT01015911), yielded only modest response in NHL and have been limited to phase I due to significant toxicities including pleural effusion, hypersensitivity and facial edema (MDX-1203), grade 3 thrombocytopenia (SGN-70A), and ocular toxicity (SGN-75) (Table 2) [101–103]. Varlilumab (CDX-1127), a human IgG1 anti-CD27 agonist, has produced substantial and durable response in the phase I trial of patients with hematologic malignancies [104].

### LAG-3

Lymphocyte activation gene-3 (LAG-3) is a transmembrane protein mainly expressed on activated CD4^+^ and CD8^+^ T cells, as well as Tregs, NK cells, and plasmacytoid dendritic cells [105]. LAG-3 and PD-1 when expressed on CD4^+^ and CD8^+^ TILs exhibit an exhausted phenotype [106]. LAG-3 blockade has been shown to synergize with anti-PD-1 blocking, suggesting LAG-3 and PD-1 signaling pathways have non-redundant and synergistic functions in dampening T cell responses within the TME [106]. LAG-3 and PD-1 double positive CD8^+^ and CD4^+^ effector T cells were coexpressed more frequently from AML BM aspirates compared with healthy donors [25]. In addition to AML, intertumoral and peripheral blood lymphocytes from HL patients also expressed high levels of LAG-3, and deletion of CD4^+^LAG-3^+^ T cells improved lymphoma-specific CD8^+^ T cell responses [107]. In CLL, both surface and soluble LAG-3 were upregulated, which were associated with a more aggressive clinical course and poor prognostic features [108]. Blocking LAG-3, but not PD-L1/PD-1 pathway, enhanced T cell activation in patients with CLL, making LAG-3 a potential target to treat CLL [108]. LAG-3 also defined the exhaustion of tumor-infiltrating PD-1^+^ T cells in B cell NHL [88, 89]. Notably, the interaction between LAG-3 and its canonical ligand, MHC-II, was unable to fully explain its suppressive functions to CD8^+^ T cells and NK cells [106]. Most recently, fibrinogen-like protein 1 (FGL1) was identified to be a major functional ligand of LAG-3 [109]. Elevated FGL1 was found in the plasma of cancer patients, and high FGL-1 level was correlated with poor prognosis and resistance to anti-PD-1 therapy [109]. It would be interesting to investigate whether FGL1/LAG-3 pathway plays a role in hematologic malignancies.

Several phase I/II clinical trials of LAG-3 antibodies as single agent (NCT03489369) or in combination with PD-1 inhibitor (NCT03005782, NCT02061761) are ongoing (Table 2). In addition, MGD013, a dual-affinity re-targeting antibody specific to both PD-1 and LAG-3 is being studied in hematologic malignancies (NCT03219268) (Table 2). As of March 2019, there are close to 27 clinical trials targeting LAG-3.

### LILRBs

The leukocyte immunoglobulin-like receptors subfamily B (LILRBs) are transmembrane glycoproteins with intracellular immunoreceptor tyrosine-based inhibitory motifs [110]. LILRB contains five members (LILRB1-5) in humans and primates, but has only two orthologs in mouse, paired immunoglobulin-like receptor B (PirB) and gp49B1, making the xenograft murine model more suitable for LILRB-related preclinical research. LILRBs are expressed on cancer cells as well as a wide range of immune cells, including NK cells, T cells, B cells, macrophages, and monocytes [110]. LILRB1 (also known as CD85J, ILT2, LIR1, and MIR7) and LILRB3 (CD85A, ILT5, LIR3, and HL9) are widely expressed on malignant cells of hematologic malignancies, such as AML, B cell leukemia/lymphoma, and T cell leukemia, where they intrinsically promote tumor progression [111]. LILRB2 (CD85D, ILT4, LIR3, and MIR10) expression was observed on human HSCs, and the binding of angiopoietin-like proteins (ANGPTLs) to LILRB2 supports ex vivo expansion of HSCs. In a transplantation AML mouse model, expression of PirB (the mouse ortholog of human LILRB2 and LILRB3) on MLL-AF9-induced AML cells was able to suppress differentiation and enhance self-renewal of LSCs [112]. It was later demonstrated that ANGPTL2/LILRB2 binding was more potent than another ligand, HLA-G [113]. LILRB4 (CD85K, ILT3, LIR5, and HM8) was restrictively expressed on monocytes and monocytic AML cells [114]. LILRB4 expression on leukemia cells suppress T-cell proliferation, as well as promote AML cell migration and infiltration. Apolipoprotein E (APOE) was identified as an extracellular binding ligand of LILRB4. APOE was able to activate LILRB4 on human monocytic AML cells, where SHP-2 was phosphorylated and NF-kB pathway was subsequently activated, resulting in the upregulation of urokinase receptor (uPAR) and arginase-1 (ARG1). As a result, ARG1 inhibited T cell proliferation, which could be augmented by uPAR signaling [114]. In addition, considering that LILRB4 was a monocytic AML-specific antigen, LILRB4-CAR-T was developed and showed efficient effector function in vitro and in vivo against LILRB4^+^ AML cells, but no toxicity to normal CD34^+^ cells [114]. As for LILRB5, its role in hematologic malignancies remains unclear [110]. Currently, there is no ongoing clinical trial evaluating LILRBs in hematologic malignancies.

### Combination of ICB with other therapies

#### Combination of ICB with bispecific T cell engager

Currently, bispecific antibodies, which recruit patient’s T cells or NK cells against cancer cells expressing tumor-associated antigens, have been attracting attention for treating hematologic malignancies. A typical example is CD33/CD3 bispecific T cell engager (BiTE). Given that CD33 is overexpressed in AML blasts, a BiTE antibody against both CD3 and CD33 has been developed to recruit T cells to kill CD33^+^ AML cells [115]. Similarly, bispecific antibody targeting both CD3 and CD123 has been designed as CD123 is overexpressed in a wide range of hematologic malignancies, particularly on LSCs [116, 117]. However, ongoing clinical trials have showed that only a small fraction of patients could benefit from bispecific antibody treatment. A major mechanism limiting the therapeutic efficacy is due to T cell anergy and exhaustion driven by inhibitory immune checkpoint pathways, such as PD-L1/PD-1 axis [118]. For example, T cells recruited to CD33-positive cells showed impaired cytotoxicity due to high expression of PD-L1 on AML cells, which was induced by CD33/CD3 BiTE antibody treatment. Inspired by the inhibitory role of PD-L1/PD-1 pathway in AML, combining PD-L1/PD-1 blockade with CD33/CD3 BiTE antibody showed enhanced T cell proliferation and IFN-γ production [119].

#### Combination of ICB with hypomethylating agents

The expression of PD-L1, PD-L2, PD-1, and CTLA-4 was upregulated in a cohort of MDS, CMML, and AML patients treated with epigenetic therapy, suggesting inhibitory immune checkpoint signaling pathways might be involved in hypomethylating agent (HMA) resistance [13]. HMAs triggered demethylation of the PD-1 promoter leading to increased expression of PD-1 on T cells, which promoted exhaustion of tumor-specific T cells and therefore resulting in immune escape [32]. Therapeutically, number of ongoing clinical trials have been designed to combine HMAs with ICB (Tables 1 and 2). Notably, AZA plus nivolumab showed better OS (16.1 months vs 4.1 months) and better ORR (33% vs 20%) in heavily treated RR AML patients compared to a historical cohort with AZA-based salvage therapy. A second cohort in this trial treated with nivolumab and ipilimumab plus AZA led to 6 of 14 patients achieving CR/CRi [32]. Responders had a progressive increase of CD4^+^ and CD8^+^ TILs in the BM, demonstrating that AML patients could benefit from PD-1 blocking therapy. Furthermore, CTLA-4^+^CD8^+^ cell numbers were increased in both responders and non-responders, indicating a dual combination of PD-1 blockade and CTLA-4 blockade with AZA might be able to further improve response rates [32].

#### Combination of ICB with cytokine therapy

Cytokines like IFN-α was approved for the treatment of hairy cell leukemia in 1986 and IL-2 for the treatment of metastatic renal cell carcinoma (1992) and advanced melanoma (1998) [120]. Although being one of the first forays in immunotherapy, nowadays, cytokine therapy is mainly used in combination with other anti-tumor treatments. For example, recently bempegaldesleukin (NKTR-214), an IL2Rβ (CD122)-biased agonist, has shown capabilities of enhancing the proliferation and activation of CD8^+^ T cells and NK cells without increasing the number of Tregs [121]. Results of PIVOT-02 trial, combination of NKTR-214 and nivolumab, has shown that this combination is safe and efficacious (ORR 48% in 23 patients) in metastatic urothelial carcinoma [122]. Aside from IL-2, IL-15 has also been evaluated in stimulating NK cells and T cells. Combination therapy with IL-15 and blocking antibodies against PD-1 and CTLA-4 has been shown to synergistically activate T cells and prolong the survival of tumor-bearing mice [123]. In addition, a recent study has demonstrated that DC-derived IL-12 is necessary for successful anti-PD-1 cancer therapy, suggesting that IL-12 and PD-1 blockade could be rationally combined [124]. In an earlier study, synergistic effects were observed when tumor-bearing mice were treated with Semliki Forest virus-based vector encoding IL-12 and anti-PD-L1 mAb [125]. Currently, there are limited pre-clinical and clinical trials based on the combination of ICB and cytokine therapy in hematologic malignancies although much more trails are ongoing in solid tumors.

## CAR-T cell immunotherapy for hematologic malignancies

CAR-T cell therapy involves genetic modification of T cells from the patient to express specific CAR, followed by ex vivo cell expansion and reinfusion back into the patient to eradicate tumors. CARs are synthetic receptors consisting of an extracellular domain, typically a single-chain variable fragment (scFv) derived from tumor antigen-reactive antibody, a transmembrane domain, and an intracellular T cell activation and co-stimulation signaling domain commonly composed of CD3ζ, CD28, and/or 4-1BB [126]. The first-generation CAR consisting of scFv attached to CD3ζ produces modest clinical results as it delivers only the first signal for T cell activation. Second-generation CARs include an additional co-stimulatory domain (CD28, 4-1BB, OX-40, and ICOS), thus enabling the CARs to deliver both signals required for full activation of T cells [126]. Third-generation CARs incorporate multiple co-stimulatory domains upstream of CD3ζ, which further enhance cytokine production and CAR-T cell persistence [126]. Fourth-generation CARs called T cell redirected for antigen-unrestricted cytokine-initiated killing (TRUCKs) encode genes for cytokine production to augment CAR-T activity or suicide genes to prevent toxicity [127]. In 2017, the US FDA approved two second-generation CAR-T cell therapies, Axicabtagene ciloleucel (axi-cel, CD3ζ-CD28) and Tisagenlecleucel (tisa-cel, CD3ζ-41bb) [128, 129]. Long-term follow-up of phase I/II ZUMA-1 clinical trial using axi-cel reported an ORR of 83% and a CR rate of 58% in RR DLBCL with durable response lasting more than 2 years [128]. Similarly for tisa-cel, the phase IIa JULIET trial produced ORR of 52% and CR rate of 40% in DLBCL patients [129]. In the interim analysis of the ELIANA phase I–II trial with tisa-cel in pediatric and adult patients with B-ALL, ORR of 81% was observed for at least 3 months after infusion. Among the patients who achieved CR, the MRD-negative remission rate was 95% by day 28 of treatment [130]. Despite the success of CD19 CAR-Ts, many technical and biological obstacles, such as toxicity, CAR-T cell dysfunction, and tumor heterogeneity and antigen loss, have limited the use of CAR-T therapy to treat other hematologic cancers and solid tumors [131]. Here, we discuss the preclinical and clinical advances of CAR-T therapies against new targets and their potential combination with ICB in treating hematologic malignancies beyond B-ALL and DLBCL.

### CD22

Although the CD19 CAR-T therapy has yielded potent antileukemic effects in children and adults with RR B-ALL, acquisition of CD19-negative cells and selection of alternatively spliced CD19 isoforms with the compromised epitope were recognized as mechanisms for tumor escape [132, 133]. Similar to CD19, CD22 (also known as Siglec-2) is also expressed on most B-ALL cells, but has a limited expression in normal tissues except B cell lineage [134, 135]. CD22 is therefore proposed as an alternative target for CAR design to treat patients with CD22-expressing B-ALL and CD19^dim^ or CD19^−^ relapse following CA19 CAR-T therapy [136]. Although CD22 CAR-T therapy demonstrated robust antileukemic activity with CR in 11 of the 15 patients and similar safety profile as CA19 CAR-T, relapse still occurred due to the loss of CD22 surface expression [136]. Importantly, a bispecific CAR targeting both CD19 and CD22 was reported to be able to overcome the resistance arising from loss of either CD19 or CD22 expression [136]. Currently, there are 17 ongoing CAR-T clinical trials targeting CD22. One particular dual specificity CD19 and CD22 CAR-T encodes truncated epidermal growth factor receptor (EGFRt) and truncated human epidermal growth factor receptor 2 (HER2t) safety switch, allowing for detection of the CAR-T cells and ADCC-directed elimination of the CAR-T cell (NCT03330691) (Table 3).

**Table 3:** Selected ongoing CAR-T trials targeting CD123, CD22, CD33, CD38, and CD138

NCT number	Phase	*n*	Conditions	Interventions	Sponsor/location
NCT03672851	I	30	RR AML	CD123 CAR-T	China
NCT03631576	II/II	20	RR AML	CD123/CLL1 CAR-T	China
NCT02937103	I/II	45	RR CD123^+^ myeloid malignancies	CD123-CAR-T	China
NCT03398967	I/II	80	RR B cell leukemia and lymphoma	Dual specificity CD19 and CD20 or CD22 CAR-T	China
NCT03330691	I	33	CD19^+^, CD22^+^ RR leukemia and lymphoma	Dual specificity CD19-HER2t CAR-T and CD22 EGFRt CAR-T	Seattle Children’s Hospital, USA
NCT03620058	I	18	RR B-ALL	CART22-65s ± huCART19	UPENN
NCT02650414	I	15	RR B-ALL	CD22 CART	UPENN, CHOP
NCT03098355	I	30	RR B-ALL or NHL	4SCAR19/22 ± interleukin-2	China
NCT03126864	I	39	RR CD33^+^ AML	CD33-CAR-T	MDACC, USA
NCT02958397	I/II	45	RR CD33^+^ myeloid malignancies	CD33-CAR-T	China
NCT03464916	I	72	RR MM	CAR2 CD38 A2 CAR-T	USA
NCT03754764	I/II	80	RR B-ALL	CD38 CAR-T after CD-19 CAR-T relapse	China
NCT03672318	I	33	RR MM	ATLCAR.CD138 CAR-T	USA
NCT03196414	I/II	10	RR MM	CART-138/BCMA CAR-T	USA
NCT03778346	I/II	30	RR MM	Fourth-generation Integrin ß7/BCMA/CS1/CD38/CD138 CAR-T or 10 different combinations	China
NCT03767751	I/II	80	RR MM	Dual CD38/BCMA CAR-T	China
NCT03222674	I/II	10	RR AML	Muc1/CLL1/CD33/CD38/CD56/CD123 CAR-T	China

*Abbreviations*: *RR* relapsed refractory, *MM* multiple myeloma, *AML* acute myeloid leukemia, *ALL* acute lymphoblastic leukemia, *NHL* non-Hodgkin’s lymphoma, *BCMA* B cell maturation antigen, *EGFRt* truncated epidermal growth factor, *HER2t* truncated human epidermal growth factor 2, *Allo-SCT* allogeneic stem cell transplantation

### CD33

CD33 (Siglec-3) is well known as a marker of myeloid progenitor cells and expressed on all normal myeloid cells [135]. Like CD22, CD33 has long been identified as a diagnostic marker and a therapeutic target for B cell lymphomas and myeloid leukemias [134]. Gemtuzumab ozogamicin (GO), a CD33-specific ADC to calicheamicin, was approved again in 2017 after being withdrawn from the market in 2010 due to safety concerns, for combination therapy with daunorubicin and cytarabine in newly diagnosed CD33^+^ AML after it doubled the event-free survival from 9.5 to 17.3 months [137]. GO is also approved as a single agent in the RR setting. Meanwhile, SGN-CD33A, another CD33 targeting ADC, was demonstrated to be more potent than GO in vitro and in a xenograft model, but the FDA called a halt to all clinical testing of SGN-CD33A after failure in a phase III trial [138]. Alternatively, CD33-specific CAR-Ts in AML are in preclinical and clinical development [139–141]. For example, CD33-CAR-T therapy exhibited potent antileukemic activities in vitro and in vivo and hematopoietic toxicity [140]. In one patient with RR CD33^+^ AML, CD33 CAR-T cell infusion led to rapid degradation of blasts in the BM within 2 weeks of infusion; however, the disease relapsed after 9 weeks as CD33^+^ blasts gradually increased. Even though the clinical toxicities observed in the patient were controllable, more patient data is needed to further validate the safety and efficacy profile of CD33 CAR-T therapy [141]. Most recently, in order to avoid potential serious adverse events caused by CD33 CAR-T therapy, a group came up with an idea to combine allogeneic transplantation of CD33 knockout (KO) HSPCs with CD33 CAR-T therapy [142]. To support this assumption, they engrafted human and rhesus macaques CD33 KO HSPCs into NSG mice and rhesus macaques model, respectively, and found that CD33 was not essential for human myeloid cell functions and rhesus macaques neutrophil functions [142]. Importantly, they demonstrated that human myeloid cells lacking expression of CD33 were resistance to CD33 CAR-T therapy in NSG mice [142]. Therapeutically, a 6-year old heavily pre-treated AML patient achieved MRD-negative remission 19 days post infusion of compound CAR (cCAR) comprising of anti-CLL1 CAR linked to anti-CD33 CAR via a self-cleaving P2A peptide [143]. Some of other ongoing CD33 CAR-T clinical trials include NCT02958397 and NCT03126864 (Table 3)

### CD123

CD123 (IL-3Rα) is normally expressed on a fraction of myeloid progenitors and a wide range of hematologic malignancies, including blastic plasmacytoid dendritic cell neoplasm (BPDCN), hairy cell leukemia, B-ALL, MDS, and AML [116, 117, 144]. Antibody-based therapies targeting CD123 have been effective in eliminating AML blasts [145]. CD123 CAR-T cells have also shown activity against CD123^+^ AML cell lines and primary patient samples in vitro and in vivo [146]. Furthermore, CD123-specific CAR cytokine-induced killer (CIK) cells had limited toxicity on normal BM HSPCs compared to CD33-specific CAR CIK cells, suggesting that CD123 CIK has a better safety profile [139]. Another group, however, raised safety concerns for the use of CD123 CAR-T due to its effect on hematopoiesis [147]. They later dementated that ablation of CAR-T cells with optimal timing after AML eradication might enable durable leukemia remission, controllable hematologic toxicity, and subsequent HSC transplantation [148]. Notably, CD123 CAR-T therapy showed remissions of AML and BPDCN, as well as acceptable feasibility and safety in the first-in-human clinical trial [149]. CD123 CAR-T therapy also exhibited specific killing activity against BPDCN and high-risk MDS in preclinical models [144, 149]. Some CD123 CAR-T trials are ongoing (Table 3).

Furthermore, a dual CAR targeting both CD19 and CD123 showed highly anti-leukemia activity against B-ALL in vivo and was able to eradicate CD19^−^ leukemic cells at relapse after CD19 CAR-T administration [150]. Treatment of 3 post allo-SCT relapse B-ALL patients with donor-derived double 4SCART19/4SCAR123 T cells helped achieve MRD-negative remission within 1 month after CAR-T infusion, without evidence of severe CRS or GvHD [151]. The pilot trial of a fourth-generation apoptosis inducible CAR targeting CD123 (CD123-scFv/CD28/CD137/CD27/CD3ζ-iCasp9) decreased disease burden from 60 to 45% in a 47-year-old patient with AML post-allo-SCT relapse [152]. CD123-CLL1 cCAR phase I clinical trial is also ongoing (Table 3).

### BCMA

B cell maturation antigen (BCMA; CD269), a member of the TNF receptor superfamily, is predominantly expressed on plasma cells and a small subset of normal B cells [153]. In patients with MM, BCMA is expressed uniformly on the surface of malignant plasma cells [154]. A novel ADC targeting BCMA has demonstrated to specifically kill MM cells without causing serious side effect, suggesting BCMA was a suitable and safe candidate for MM treatment [153]. BCMA-specific CAR-T cells have shown effective depletion of MM cells both in vitro and in vivo [155].

Clinical data over the past 2 years with BCMA-specific CAR-T cells has produced MRD-negative remission in heavily pre-treated MM patients [156–159]. NCI published the first-in-humans clinical trial and reported ORR of 81% and a very good partial response (VGPR) of 63% in RR MM patients with median number of 10 prior therapies [156]. The bb2121 CAR-T (Bluebird Bio) produced ORR of 85%, median DOR of 10.9 months, and median PFS of 11.8 months in 33 heavily pretreated (median number of 7 prior therapies; range 3-23) in the phase I, CRB-401 clinical trial [159]. Further, 45% achieved CR (*n* = 15), 9% achieved stringer CR, and 27% achieved VGPR. Sixteen patients achieved MRD negative remission and the median time to at least a PR was 1 month [159]. The LCAR-B38M CAR-T (LEGEND) uses a new antigen-binding domain that binds to two different antigen epitopes and reported ORR of 88% in 57 patients and MRD-negative remission in 39 of 42 patients in complete remission [158]. Two other abstracts presented by the Memorial Sloan Kettering group at American Society of Hematology annual meeting (ASH 2018) reported ORRs of 64% and 82% with the MCARH171 and JCAR125 CAR-T cells, respectively. The MCARH171 CAR-T encodes for the truncated epidermal growth factor receptor safety system [160]. The University of Pennsylvania CART-BCMA demonstrated ORR of 62% in patients with high-risk cytogenetics including 67% with TP53 or del17p mutation. In vivo CAR-T expansion was higher with the use of cyclophosphamide conditioning and a trend towards benefit was observed with higher peak CAR-T levels although this was not statistically significant [157]. BCMA-targeted CAR-Ts have produced impressive results thus far. However, the durability of the responses remains to be explored.

### CD38

CD38 is a type II transmembrane glycoprotein associated with cell-surface receptors in lipid rafts and is able to induce cell growth signal in myeloid leukemia [161]. CD38 is highly and consistently expressed on MM cells and is absent on normal myeloid and lymphoid cells, as well as other nonhematopoietic tissues [161, 162]. Several modified anti-CD38 mAbs, such as daratumumab, isatuximab, and MOR202, have been developed to treat CD38+ RR MM via mechanisms of action including Fc-dependent immune-effector manner and immunomodulatory effects [161, 163, 164]. Of note, daratumumab was approved by the FDA in 2015 to treat MM patients who had received at least three prior lines of therapy. In the presence of rituximab, combining anti-CD19 and anti-CD38 CARs showed synergistic cytotoxicity against B-NHL in vitro and in xenograft mice, providing a powerful rationale for clinical evaluation of CD38 CAR and/or CD19 CAR in the treatment of patients with relapsed B-NHLs after rituximab therapy [165]. However, with high-affinity CD38 CAR-T, off-target toxicities were also observed in addition to expected anti-MM effects. To address the safety concerns, a CAR with lower affinity anti-CD38 scFv was designed. It exhibited better discriminative capacity between MM cells and normal cells without significant loss of expansion, persistence, and cytotoxic potential [166]. Another attempt of CD38 CAR-T optimization utilized “light-chain exchange” technology, which generates new antibodies with up to 1000-fold lower affinities to CD38. By incorporation of scFv with different affinities, high-affinity and low-affinity CD38 CAR-Ts were made. As predicted, low-affinity CD38 CAR-T cells had similar effects as high-affinity CD38 CAR-T cells in eradicating MM cell line UM9, while showed no obvious effect on normal HSPCs in vivo [166].

Clinical trials with CD38 CAR-T in RR MM (NCT03464916) and RR B-ALL (NCT03754764) are underway. In addition, dual specificity CD38/BCMA CAR-T (NCT03767751) is also being explored (Table 3).

### CD138

CD138 (Syndecan-1) is a membrane glycoprotein expressed on malignant and healthy differentiated plasma cells, as well as in normal and neoplastic epithelial tissues [167]. CD138 is one of the most specific primary diagnostic markers of MM [162]. A phase I/IIa study in MM patients showed that CD138-specific ADC was well tolerated, suggesting CD138 was a targetable MM-specific antigen [168]. Importantly, in a pilot clinical trial evaluating CD138-directed CAR-T therapy, 4 out of 5 patients diagnosed with chemotherapy-refractory MM experienced myeloma regression and had stable disease longer than 3 months. The study suggests that CD138 CAR-T is safe and tolerable [169]. Dual CD138 and BCMA as well as multi-target CAR-T trials NCT03672318, NCT03196414, NCT03778346 are ongoing (Table 3).

### Combination of CAR-T and ICB in hematologic malignancies

Despite the encouraging outcomes of CD19 CAR-T therapy in B cell malignancies, poor T cell expansion and short-term T cell persistence remain one of the main causes for lack of response and relapse following CAR-T therapy. Development of T cell exhaustion induced by co-inhibitory pathways has been suspected to contribute to poor persistence and dysfunctions of CAR-T cells [170]. In order to understand why only 26% of CLL patients benefited from CD19 CAR-T therapy while over 90% of CD19-positive B-ALL experienced CR, a detailed transcriptomic analysis was performed to compare T cells from CLL responders and non-responders post CD19 CAR-T therapy. It revealed that CAR-T cells from non-responders showed upregulated pathways involved in exhaustion and apoptosis [130, 171]. The expression level of T cell co-inhibitory receptors, such as PD-1, Tim-3, and LAG-3, were upregulated on CAR-T cells, suggesting possible inhibitory effects induced by these molecules [172, 173]. The PD-L1/PD-1 pathway was able to directly inactivate CD28 signaling in CAR-T using CD28 as co-stimulatory domain and therefore inhibiting CAR-T cell function [173, 174]. Furthermore, PD-1 or LAG-3-deficient CAR-T cells showed improved anti-tumor efficacy in vitro and in vivo [175]. The addition of PD-1 blockade to CD19 CAR-T therapy in 14 children (13 with pembrolizumab and 1 with nivolumab) with heavily pre-treated B-ALL including allo-SCT who initially had poor response to CD19 CAR-T therapy had improved persistence of CAR-T cells, thus resulting in better outcomes in this small, single-center study at Children’s Hospital of Pennsylvania (CHOP). Seven of the 14 patients maintained either PR or CR. Three of 6 patients treated with PD-1 inhibitor re-established B cell aplasia suggesting ongoing CAR-T function [176].

## Conclusion

ICB with PD-1/PDL-/CTLA4 inhibitors and CAR-T therapy targeting CD19^+^ leukemia/lymphoma have forever changed the landscape of cancer therapeutics. The identification of novel immune checkpoints will fill in the gap in which our current therapeutics do not work or after disease relapse. CAR-T therapy has expanded beyond CD19^+^ with newer targets, and the engineering has become safer and sophisticated with the introduction of cytokines or safety switches. Dual specificity CAR-Ts combat disease relapse due to antigen loss, and the combination of ICB and CAR-T also has shown enhanced therapeutic efficacy. Much remains to be investigated about the optimal method of administrating the new CAR-Ts, their safety, and durability of response. However, as we garner a better understanding of the interplay between these targets and their mechanism of action, the field of immune therapy has the potential to reach more patients and transform cancer care.

## Data Availability

Not applicable

## Abbreviations

ABVD: Adriamycin, bleomycin, vinblastine, dacarbazine
ADC: Antibody-drug conjugate
ADCC: Antibody-dependent cellular cytotoxicity
ALL: Acute lymphoid leukemia
Allo-SCT: Allogenic stem cell transplantation
AML: Acute myeloid leukemia
ANGPTLs: Angiopoietin-like proteins
APC: Antigen-presenting cells
APOE: Apolipoprotein E
ARDS: Acute respiratory distress syndrome
ARG1: Arginase-1
ASH 2018: American Society of Hematology annual meeting 2018
Auto-SCT: Autologous stem cell transplantation
AZA: Azacytidine
BCMA: B cell maturation antigen
BiTE: Bispecific T cell engager
BM: Bone marrow
BPDCN: Blastic plasmacytoid dendritic cell neoplasm
BV: Brentuximab vedotin
CAR: Chimeric antigen receptor
cCAR: Compound CAR
CIK: Cytokine-induced killer
CLL: Chronic lymphocytic leukemia
CML: Chronic myeloid leukemia
CR: Complete remission
CTL: Cytotoxic T lymphocyte
CTLA-4: Cytotoxic T lymphocyte-associated protein 4
DLBCL: Diffuse large B cell lymphoma
DOR: Duration of response
EGFRt: Truncated epidermal growth factor
FGL1: Fibrinogen-like protein 1
FL: Follicular lymphoma
FLT-3: FMS-like tyrosine kinase 3
GCB: Germinal central B cell
GO: Gemtuzumab ozogamicin
GvHD: Graft versus host disease
HER2t: Truncated human epidermal growth factor receptor 2
HL: Hodgkin’s lymphoma
HMA: Hypomethylating agent
HSC: Hematopoietic stem cell
ICB: Immune checkpoint blockade
IFN: Interferon
IL: Interleukin
IMiDs: Immunomodulatory drugs
irAE: Immune-related adverse events
JAK: Janus kinase
KO: Knock out
LAG-3: Lymphocyte activation gene-3
LILRB: Leukocyte immunoglobulin-like receptors subfamily B
LSC: Leukemia stem cell
mAb: Monoclonal antibody
MDS: Myelodysplastic syndrome
MEK/ERK: Extracellular signal-regulated kinase
MHC: Major histocompatibility complex
MM: Multiple myeloma
MRD: Minimal residual disease
NHL: Non-Hodgkin’s lymphoma
ORR: Overall response rates
OS: Overall survival
PC: Plasma cells
PD-1: Program cell death protein 1
PD-L1: Programmed cell death ligand 1
PD-L2: Programmed cell death ligand 2
PFS: Progression-free survival
PirB: Paired immunoglobulin-like receptor B
PMBCL: Primary mediastinal B cell lymphoma
PR: Partial response
PTCL: Peripheral T cell lymphoma
RR: Relapsed/refractory
RS: Reed Sternberg
SD: Stable disease
SLL: Small lymphocytic leukemia
TCR: T cell receptor
TILs: Tumor-infiltrating lymphocytes
Tim-3: T cell immunoglobulin and mucin-domain containing-3
TME: Tumor microenvironment
Tregs: T regulatory cells
TRUCKs: T cells redirected for antigen-unrestricted cytokine-initiated killing
uPAR: Urokinase receptor
VGPR: Very good partial response

## References

[1] Morrison SJ, Scadden DT (2014). The bone marrow niche for haematopoietic stem cells. Nature.

[2] Orkin SH, Zon LI (2008). Hematopoiesis: an evolving paradigm for stem cell biology. Cell.

[3] Siegel RL, Miller KD, Jemal A (2019). Cancer statistics, 2019. Ca-a Cancer Journal for Clinicians.

[4] Eppert K, Takenaka K, Lechman ER, Waldron L, Nilsson B, van Galen P (2011). Stem cell gene expression programs influence clinical outcome in human leukemia. Nature Medicine.

[5] Lafferty KJ, Gill RG (1993). The maintenance of self-tolerance. Immunology and Cell Biology.

[6] Janakiram M, Chinai JM, Fineberg S, Fiser A, Montagna C, Medavarapu R (2015). Expression, clinical significance, and receptor identification of the newest B7 family member HHLA2 protein. Clinical cancer research.

[7] Leach DR, Krummel MF, Allison JP (1996). Enhancement of antitumor immunity by CTLA-4 blockade. Science.

[8] Hodi FS, O'Day SJ, McDermott DF, Weber RW, Sosman JA, Haanen JB (2010). Improved survival with ipilimumab in patients with metastatic melanoma. New England Journal of Medicine.

[9] Zang XX (2018). 2018 Nobel Prize in medicine awarded to cancer immunotherapy: immune checkpoint blockade-A personal account. Genes & Diseases.

[10] Le Dieu R, Taussig DC, Ramsay AG, Mitter R, Miraki-Moud F, Fatah R (2009). Peripheral blood T cells in acute myeloid leukemia (AML) patients at diagnosis have abnormal phenotype and genotype and form defective immune synapses with AML blasts. Blood.

[11] Pistillo MP, Tazzari PL, Palmisano GL, Pierri I, Bolognesi A, Ferlito F (2003). CTLA-4 is not restricted to the lymphoid cell lineage and can function as a target molecule for apoptosis induction of leukemic cells. Blood.

[12] Laurent S, Palmisano GL, Martelli AM, Kato T, Tazzari PL, Pierri I (2007). CTLA-4 expressed by chemoresistant, as well as untreated, myeloid leukaemia cells can be targeted with ligands to induce apoptosis. British Journal of Haematology.

[13] Yang H, Bueso-Ramos C, DiNardo C, Estecio MR, Davanlou M, Geng QR (2014). Expression of PD-L1, PD-L2, PD-1 and CTLA4 in myelodysplastic syndromes is enhanced by treatment with hypomethylating agents. Leukemia.

[14] LaBelle JL, Hanke CA, Blazar BR, Truitt RL (2002). Negative effect of CTLA-4 on induction of T-cell immunity in vivo to B7-1(+), but not B7-2(+), marine myelogenous leukemia. Blood.

[15] Saudemont A, Quesnel B (2004). In a model of tumor dormancy, long-term persistent leukemic cells have increased B7-H1 and B7.1 expression and resist CTL-mediated lysis. Blood.

[16] Huurman VAL, Unger WWJ, Koeleman BPC, Oaks MK, Chandraker AK, Terpstra OT (2007). Differential inhibition of autoreactive memory- and alloreactive naive T cell responses by soluble cytotoxic T lymphocyte antigen 4 (sCTLA4), CTLA4Ig and LEA29Y. Clinical and Experimental Immunology.

[17] Marshall NA, Christie LE, Munro LR, Culligan DJ, Johnston PW, Barker RN (2004). Immunosuppressive regulatory T cells are abundant in the reactive lymphocytes of Hodgkin lymphoma. Blood.

[18] Motta M, Rassenti L, Shelvin BJ, Lerner S, Kipps TJ, Keating MJ (2005). Increased expression of CD152 (CTLA-4) by normal T lymphocytes in untreated patients with B-cell chronic lymphocytic leukemia. Leukemia.

[19] Do P, Beckwith KA, Beaver L, Griffin BG, Mo XK, Jones J (2016). Leukemic cell expressed CTLA-4 suppresses T cells via down-modulation of CD80 by trans-endocytosis. Blood.

[20] Monne M, Piras G, Palmas A, Arru L, Murineddu M, Latte G (2004). Cytotoxic T-lymphocyte antigen-4 (CTLA-4) gene polymorphism and susceptibility to non-Hodgkin’s lymphoma. American Journal of Hematology.

[21] Braga WMT, da Silva BR, de Carvalho AC, Maekawa YH, Bortoluzzo AB, Rizzatti EG (2014). FOXP3 and CTLA4 overexpression in multiple myeloma bone marrow as a sign of accumulation of CD4(+) T regulatory cells. Cancer Immunology Immunotherapy.

[22] Zhang L, Gajewski TF, Kline J (2009). PD-1/PD-L1 interactions inhibit antitumor immune responses in a murine acute myeloid leukemia model. Blood.

[23] Dail M, Yang L, Green C, Ma C, Robert A, Kadel EE (2016). Distinct patterns of PD-L1 and PD-L2 expression by tumor and non-tumor cells in patients with MM, MDS and AML. Blood.

[24] Liakou CI, Kamat A, Tang DN, Chen H, Sun JJ, Troncoso P (2008). CTLA-4 blockade increases IFN gamma-producing CD4(+)ICOS(hi) cells to shift the ratio of effector to regulatory T cells in cancer patients. Proceedings of the National Academy of Sciences of the United States of America.

[25] Williams P, Basu S, Garcia-Manero G, Hourigan CS, Oetjen KA, Cortes JE (2019). The distribution of T-cell subsets and the expression of immune checkpoint receptors and ligands in patients with newly diagnosed and relapsed acute myeloid leukemia. Cancer.

[26] Yang SM, Huang XJ (2016). The poorer-risk AML, the weaker immunologic surveillance? Higher PD-L1 expression on non-APL AML cells is associated with poorer risk status according to cytogenetics and molecular abnormalities. Blood.

[27] Berthon C, Driss V, Liu JZ, Kuranda K, Leleu X, Jouy N (2010). In acute myeloid leukemia, B7-H1 (PD-L1) protection of blasts from cytotoxic T cells is induced by TLR ligands and interferon-gamma and can be reversed using MEK inhibitors. Cancer Immunology Immunotherapy.

[28] Daver N, Basu S, Garcia-Manero G, Cortes JE, Ravandi F, Ning J (2016). Defining the immune checkpoint landscape in patients (pts) with acute myeloid leukemia (AML). Blood.

[29] Schnorfeil FM, Lichtenegger FS, Emmerig K, Schlueter M, Neitz JS, Draenert R (2015). T cells are functionally not impaired in AML: increased PD-1 expression is only seen at time of relapse and correlates with a shift towards the memory T cell compartment. Journal of Hematology & Oncology.

[30] Mumprecht S, Schurch C, Schwaller J, Solenthaler M, Ochsenbein AF (2009). Programmed death 1 signaling on chronic myeloid leukemia-specific T cells results in T-cell exhaustion and disease progression. Blood.

[31] Davids MS, Kim HT, Bachireddy P, Costello C, Liguori R, Savell A (2016). Ipilimumab for patients with relapse after allogeneic transplantation. New England Journal of Medicine.

[32] Daver NG, Garcia-Manero G, Basu S, Cortes JE, Ravandi F, Kadia TM (2018). Safety, efficacy, and biomarkers of response to azacitidine (AZA) with nivolumab (Nivo) and AZA with nivo and ipilimumab (Ipi) in relapsed/refractory acute myeloid leukemia: a non-randomized, phase 2 study. Blood.

[33] Kadia TM, Cortes JE, Ghorab A, Ravandi F, Jabbour E, Daver NG (2018). Nivolumab (Nivo) maintenance (maint) in high-risk (HR) acute myeloid leukemia (AML) patients. Journal of Clinical Oncology.

[34] Zeidner JF, Vincent BG, Ivanova A, Foster MC, Coombs CC, Jamieson K (2018). Genomics reveal potential biomarkers of response to pembrolizumab after high dose cytarabine in an ongoing phase II trial in relapsed/refractory AML. Blood.

[35] Ravandi F, Daver N, Garcia-Manero G, Benton CB, Thompson PA, Borthakur G (2017). Phase 2 study of combination of cytarabine, idarubicin, and nivolumab for initial therapy of patients with newly diagnosed acute myeloid leukemia. Blood.

[36] Assi R, Kantarjian HM, Daver NG, Garcia-Manero G, Benton CB, Thompson PA (2018). Results of a phase 2, open-label study of idarubicin (I), cytarabine (A) and nivolumab (Nivo) in patients with newly diagnosed acute myeloid leukemia (AML) and high-risk myelodysplastic syndrome (MDS). Blood.

[37] Ansell S, Armand P, Timmerman JM, Shipp MA, Bradley Garelik MB, Zhu L (2015). Nivolumab in patients (Pts) with relapsed or refractory classical Hodgkin lymphoma (R/R cHL): clinical outcomes from extended follow-up of a phase 1 study (CA209-039). Blood.

[38] Ansell SM, Lesokhin AM, Borrello I, Halwani A, Scott EC, Gutierrez M (2015). PD-1 blockade with nivolumab in relapsed or refractory Hodgkin’s lymphoma. New England Journal of Medicine.

[39] Lesokhin AM, Ansell SM, Armand P, Scott EC, Halwani A, Gutierrez M (2016). Nivolumab in patients with relapsed or refractory hematologic malignancy: preliminary results of a phase Ib study. Journal of clinical oncology.

[40] Ansell S, Gutierrez ME, Shipp MA, Gladstone D, Moskowitz A, Borello I (2016). A phase 1 study of nivolumab in combination with ipilimumab for relapsed or refractory hematologic malignancies (CheckMate 039). Blood.

[41] Armand P, Engert A, Younes A, Fanale M, Santoro A, Zinzani PL (2018). Nivolumab for relapsed/refractory classic Hodgkin lymphoma after failure of autologous hematopoietic cell transplantation: extended follow-up of the multicohort single-arm phase II CheckMate 205 trial. Journal of Clinical Oncology.

[42] Younes A, Santoro A, Shipp M, Zinzani PL, Timmerman JM, Ansell S (2016). Nivolumab for classical Hodgkin’s lymphoma after failure of both autologous stem-cell transplantation and brentuximab vedotin: a multicentre, multicohort, single-arm phase 2 trial. Lancet Oncology.

[43] Armand P, Shipp MA, Ribrag V, Michot JM, Zinzani PL, Kuruvilla J (2016). Programmed death-1 blockade with pembrolizumab in patients with classical Hodgkin lymphoma after brentuximab vedotin failure. Journal of Clinical Oncology.

[44] Armand P (2018). Pembrolizumab in patients with relapsed or refractory primary mediastinal large B-cell lymphoma (PMBCL): data from the Keynote-013 and Keynote-170 studies. Blood.

[45] Chen R, Zinzani PL, Fanale MA, Armand P, Johnson NA, Brice P (2017). Phase II study of the efficacy and safety of pembrolizumab for relapsed/refractory classic Hodgkin lymphoma. Journal of Clinical Oncology.

[46] Zinzani PL (2018). Two-year follow-up of Keynote-087 Study: pembrolizumab monotherapy in relapsed/refractory classic Hodgkin lymphoma. Blood.

[47] Herrera AF, Moskowitz AJ, Bartlett NL, Vose JM, Ramchandren R, Feldman TA (2018). Interim results of brentuximab vedotin in combination with nivolumab in patients with relapsed or refractory Hodgkin lymphoma. Blood.

[48] Diefenbach C (2018). A phase I study with an expansion cohort of the combinations of ipilimumab, nivolumab and brentuximab vedotin in patients with relapsed/refractory Hodgkin lymphoma: a trial of the ECOG-ACRIN research group (E4412: Arms G-I). Blood.

[49] Wong E, Dawson E, Davis J, Koldej R, Ludford-Menting M, Lansdown M (2018). Nivolumab for relapsed or residual haematological malignancies after allogeneic haematopoietic stem cell transplantation (NIVALLO). Blood.

[50] Davids MS, Kim HT, Costello CL, Herrera AF, Locke FL, Maegawa RO (2018). A phase I/Ib study of nivolumab for relapsed hematologic malignancies after allogeneic hematopoietic cell transplantation (alloHCT). Blood.

[51] Li XF, Deng RS, He W, Liu C, Wang M, Young J (2012). Loss of B7-H1 expression by recipient parenchymal cells leads to expansion of infiltrating donor CD8(+) T cells and persistence of graft-versus-host disease. Journal of Immunology.

[52] Michonneau D, Sagoo P, Breart B, Garcia Z, Celli S, Bousso P (2016). The PD-1 axis enforces an anatomical segregation of CTL activity that creates tumor niches after allogeneic hematopoietic stem cell transplantation. Immunity.

[53] Yamamoto R, Nishikori M, Tashima M, Sakai T, Ichinohe T, Takaori-Kondo A (2009). B7-H1 expression is regulated by MEK/ERK signaling pathway in anaplastic large cell lymphoma and Hodgkin lymphoma. Cancer Science.

[54] Green MR, Monti S, Rodig SJ, Juszczynski P, Currie T, O’Donnell E (2010). Integrative analysis reveals selective 9p24.1 amplification, increased PD-1 ligand expression, and further induction via JAK2 in nodular sclerosing Hodgkin lymphoma and primary mediastinal large B-cell lymphoma. Blood.

[55] Muenst S, Hoeller S, Dirnhofer S, Tzankov A (2009). Increased programmed death-1+ tumor-infiltrating lymphocytes in classical Hodgkin lymphoma substantiate reduced overall survival. Human Pathology.

[56] Xu-Monette ZY, Zhou JF, Young KH (2018). PD-1 expression and clinical PD-1 blockade in B-cell lymphomas. Blood.

[57] Yamamoto R, Nishikori M, Kitawaki T, Sakai T, Hishizawa M, Tashima M (2008). PD-1-PD-1 ligand interaction contributes to immunosuppressive microenvironment of Hodgkin lymphoma. Blood.

[58] Vari F, Arpon D, Keane C, Hertzberg MS, Talaulikar D, Jain S (2018). Immune evasion via PD-1/PD-L1 on NK cells and monocyte/macrophages is more prominent in Hodgkin lymphoma than DLBCL. Blood.

[59] Cader FZ, Schackmann RCJ, Hu XH, Wienand K, Redd R, Chapuy B (2018). Mass cytometry of Hodgkin lymphoma reveals a CD4(+) regulatory T-cell-rich and exhausted T-effector microenvironment. Blood.

[60] Andorsky DJ, Yamada RE, Said J, Pinkus GS, Betting DJ, Timmerman JM (2011). Programmed death ligand 1 is expressed by non-Hodgkin lymphomas and inhibits the activity of tumor-associated T cells. Clinical cancer research.

[61] Kiyasu J, Miyoshi H, Hirata A, Arakawa F, Ichikawa A, Niino D (2015). Expression of programmed cell death ligand 1 is associated with poor overall survival in patients with diffuse large B-cell lymphoma. Blood.

[62] Goodman A, Patel SP, Kurzrock R (2017). PD-1–PD-L1 immune-checkpoint blockade in B-cell lymphomas. Nature Reviews Clinical Oncology.

[63] Myklebust JH, Irish JM, Brody J, Czerwinski DK, Houot R, Kohrt HE (2013). High PD-1 expression and suppressed cytokine signaling distinguish T cells infiltrating follicular lymphoma tumors from peripheral T cells. Blood.

[64] Carreras J, Lopez-Guillermo A, Roncador G, Villamor N, Colomo L, Martinez A (2009). High numbers of tumor-infiltrating programmed cell death 1-positive regulatory lymphocytes are associated with improved overall survival in follicular lymphoma. Journal of Clinical Oncology.

[65] Xerri L, Chetailie B, Seriari N, Attias C, Guillaume Y, Arnoulet C (2008). Programmed death 1 is a marker of angioimmunoblastic T-cell lymphoma and B-cell small lymphocytic lymphoma/chronic lymphocytic leukemia. Human Pathology.

[66] Brusa D, Serra S, Coscia M, Rossi D, D'Arena G, Laurenti L (2013). The PD-1/PD-L1 axis contributes to T-cell dysfunction in chronic lymphocytic leukemia. Haematologica.

[67] Gorgun G, Samur MK, Cowens KB, Paula S, Bianchi G, Anderson JE (2015). Lenalidomide enhances immune checkpoint blockade-induced immune response in multiple myeloma. Clinical cancer research.

[68] Paiva B, Azpilikueta A, Puig N, Ocio EM, Sharma R, Oyajobi BO (2015). PD-L1/PD-1 presence in the tumor microenvironment and activity of PD-1 blockade in multiple myeloma. Leukemia.

[69] Benson DM, Bakan CE, Mishra A, Hofmeister CC, Efebera Y, Becknell B (2010). The PD-1/PD-L1 axis modulates the natural killer cell versus multiple myeloma effect: a therapeutic target for CT-011, a novel monoclonal anti-PD-1 antibody. Blood.

[70] Ray A, Das DS, Song Y, Richardson P, Munshi NC, Chauhan D (2015). Targeting PD1-PDL1 immune checkpoint in plasmacytoid dendritic cell interactions with T cells, natural killer cells and multiple myeloma cells. Leukemia.

[71] Luptakova K, Rosenblatt J, Glotzbecker B, Mills H, Stroopinsky D, Kufe T (2013). Lenalidomide enhances anti-myeloma cellular immunity. Cancer Immunology Immunotherapy.

[72] Ansell SM, Minnema MC, Johnson P, Timmerman JM, Armand P, Shipp MA (2019). Nivolumab for relapsed/refractory diffuse large B-cell lymphoma in patients ineligible for or having failed autologous transplantation: a single-arm, phase II study. Journal of Clinical Oncology.

[73] Chen Y-B (2018). PD-1 blockade for diffuse large B-cell lymphoma after autologous stem cell transplantation. Blood.

[74] Nastoupil Loretta J., Westin Jason R., Fowler Nathan Hale, Fanale Michelle A., Samaniego Felipe, Oki Yasuhiro, Obi Chizobam, Cao JingJing, Cheng Xiaoyun, Ma Man Chun John, Wang Zhiqiang, Chu Fuliang, Feng Lei, Zhou Shouhao, Davis Richard Eric, Neelapu Sattva Swarup (2017). Response rates with pembrolizumab in combination with rituximab in patients with relapsed follicular lymphoma: Interim results of an on open-label, phase II study. Journal of Clinical Oncology.

[75] Younes A, Brody J, Carpio C, Lopez-Guillermo A, Ben-Yehuda D, Ferhanoglu B (2019). Safety and activity of ibrutinib in combination with nivolumab in patients with relapsed non-Hodgkin lymphoma or chronic lymphocytic leukaemia: a phase 1/2a study. The Lancet Haematology.

[76] Ocio Enrique M., Mateos Maria-Victoria, Orlowski Robert Z., Siegel David, Reece Donna Ellen, Moreau Philippe, Rodriguez-Otero Paula, Munshi Nikhil C., Avigan David, Ghori Razi, Wnek Richard, Mogg Robin, Marinello Patricia Maria, San Miguel Jesus (2017). Pembrolizumab (Pembro) plus lenalidomide (Len) and low-dose dexamethasone (Dex) for relapsed/refractory multiple myeloma (RRMM): Efficacy and biomarker analyses. Journal of Clinical Oncology.

[77] Pawarode A, D'Souza A, PM C, Johnson B, Braun T, Dhakal B (2017). Phase 2 study of pembrolizumab during lymphodepleted state after autologous hematopoietic cell transplantation in multiple myeloma patients. Blood.

[78] Biran N, Andrews T, Feinman R, Vesole DH, Richter JR, Zenreich J (2017). A phase II trial of the anti-PD-1 monoclonal antibody pembrolizumab (MK-3475) + lenalidomide + dexamethasone as post autologous stem cell transplant consolidation in patients with high-risk multiple yeloma. Blood.

[79] Mateos Maria-Victoria, Blacklock Hilary, Schjesvold Fredrik, Rocafiguera Albert Oriol, Simpson David, George Anupkumar, Goldschmidt Hartmut, Larocca Alessandra, Sherbenou Daniel Wayne, Avivi Irit, Iida Shinsuke, Matsumoto Morio, Usmani Saad Zafar, Jagannath Sundar, Rodriguez-Otero Paula, Kher Uma, Farooqui Mohammed Z.H., Liao Jason, Marinello Patricia, Lonial Sagar (2018). A phase 3 randomized study of pembrolizumab (Pembro) plus pomalidomide (Pom) and dexamethasone (Dex) for relapsed/refractory multiple myeloma (RRMM): KEYNOTE-183. Journal of Clinical Oncology.

[80] Chen RW, Ansell SM, Zinzani PL, Vacirca JL, Lopez-Guillermo A, Hutchings M (2017). Phase 1b/3 study of avelumab-based combination regimens in patients with relapsed or refractory diffuse large B-cell lymphoma (R/R DLBCL). Journal of Clinical Oncology.

[81] Sakuishi K, Apetoh L, Sullivan JM, Blazar BR, Kuchroo VK, Anderson AC (2010). Targeting Tim-3 and PD-1 pathways to reverse T cell exhaustion and restore anti-tumor immunity. Journal of Experimental Medicine.

[82] Zhu C, Anderson AC, Schubart A, Xiong HB, Imitola J, Khoury SJ (2005). The Tim-3 ligand galectin-9 negatively regulates T helper type 1 immunity. Nature Immunology.

[83] Kong Y, Zhang J, Claxton DF, Ehmann WC, Rybka WB, Zhu L (2015). PD-1(hi)TIM-3(+) T cells associate with and predict leukemia relapse in AML patients post allogeneic stem cell transplantation. Blood Cancer Journal.

[84] Kikushige Y, Shima T, Takayanagi S, Urata S, Miyamoto T, Iwasaki H (2010). TIM-3 is a promising target to selectively kill acute myeloid leukemia stem cells. Cell Stem Cell.

[85] Silva IG, Yasinska IM, Sakhnevych SS, Fiedler W, Wellbrock J, Bardelli M (2017). The Tim-3-galectin-9 secretory pathway is involved in the immune escape of human acute myeloid leukemia cells. Ebiomedicine.

[86] Prokhorov A, Gibbs BF, Bardelli M, Ruegg L, Fasler-Kan E, Varani L (2015). The immune receptor Tim-3 mediates activation of PI3 kinase/mTOR and HIF-1 pathways in human myeloid leukaemia cells. International Journal of Biochemistry & Cell Biology.

[87] Kikushige Y, Miyamoto T, Yuda J, Jabbarzadeh-Tabrizi S, Shima T, Takayanagi S (2015). A TIM-3/Gal-9 autocrine stimulatory loop drives self-renewal of human myeloid leukemia stem cells and leukemic progression. Cell Stem Cell.

[88] Yang ZZ, Price-troska T, Novak AJ, Ansell SM (2015). The exhausted intratumoral T cell population in B-cell non-Hodgkin lymphoma is defined by LAG-3, PD-1 and Tim-3 expression. Blood.

[89] Dashnamoorthy R, Chen B, Galera P, Chang H, Beheshti A, Ghosh S (2017). The immune checkpoint receptors PD-1, PD-L1, TIM-3 and LAG-3 in lymphoma: tumor cell and tumor infiltrating lymphocyte (TIL) expression, patient prognostication, and identification of rational therapeutic targets. Blood.

[90] Hendriks J, Gravestein LA, Tesselaar K, van Lier RAW, Schumacher TNM, Borst J (2000). CD27 is required for generation and long-term maintenance of T cell immunity. Nature Immunology.

[91] Gattinoni L, Lugli E, Ji Y, Pos Z, Paulos CM, Quigley MF (2011). A human memory T cell subset with stem cell-like properties. Nature Medicine.

[92] Nolte MA, Arens R, van Os R, van Oosterwijk M, Hooibrink B, van Lier RAW (2005). Immune activation modulates hematopoiesis through interactions between CD27 and CD70. Nature Immunology.

[93] Schurch C, Riether C, Matter MS, Tzankov A, Ochsenbein AF (2012). CD27 signaling on chronic myelogenous leukemia stem cells activates Wnt target genes and promotes disease progression. Journal of Clinical Investigation.

[94] Riether C, Schurch CM, Flury C, Hinterbrandner M, Druck L, Huguenin AL (2015). Tyrosine kinase inhibitor-induced CD70 expression mediates drug resistance in leukemia stem cells by activating Wnt signaling. Science Translational Medicine.

[95] Riether C, Schurch CM, Buhrer ED, Hinterbrandner M, Huguenin AL, Hoepner S (2017). CD70/CD27 signaling promotes blast stemness and is a viable therapeutic target in acute myeloid leukemia. Journal of Experimental Medicine.

[96] He LZ, Thomas L, Weidlick J, Vitale L, O'Neill T, Prostak N (2011). Development of a human anti-CD27 antibody with efficacy in lymphoma and leukemia models by two distinct mechanisms. Blood.

[97] Grewal IS (2008). CD70 as a therapeutic target in human malignancies. Expert Opinion on Therapeutic Targets.

[98] Yang ZZ, Grote DM, Xiu B, Ziesmer SC, Price-Troska TL, Hodge LS (2014). TGF-beta upregulates CD70 expression and induces exhaustion of effector memory T cells in B-cell non-Hodgkin's lymphoma. Leukemia.

[99] Al Sayed MF, Ruckstuhl CA, Hilmenyuk T, Claus C, Bourquin JP, Bornhauser BC (2017). CD70 reverse signaling enhances NK cell function and immunosurveillance in CD27-expressing B-cell malignancies. Blood.

[100] Bagot M (2018). Argx-110 for treatment of CD70-positive advanced cutaneous T-cell lymphoma in a phase 1/2 clinical trial. Blood.

[101] Tannir NM, Forero-Torres A, Ramchandren R, Pal SK, Ansell SM, Infante JR (2014). Phase I dose-escalation study of SGN-75 in patients with CD70-positive relapsed/refractory non-Hodgkin lymphoma or metastatic renal cell carcinoma. Investigational New Drugs.

[102] Owonikoko TK, Hussain A, Stadler WM, Smith DC, Kluger H, Molina AM (2016). First-in-human multicenter phase I study of BMS-936561 (MDX-1203), an antibody-drug conjugate targeting CD70. Cancer Chemotherapy and Pharmacology.

[103] Phillips Tycel, Barr Paul M., Park Steven I., Kolibaba Kathryn, Caimi Paolo F., Chhabra Saurabh, Kingsley Edwin C., Boyd Thomas, Chen Robert, Carret Anne-Sophie, Gartner Elaina M., Li Hong, Yu Cindy, Smith David C. (2018). A phase 1 trial of SGN-CD70A in patients with CD70-positive diffuse large B cell lymphoma and mantle cell lymphoma. Investigational New Drugs.

[104] Ansell Stephen Maxted, Northfelt Donald W., Flinn Ian, Burris Howard A., Dinner Shira Naomi, Villalobos Victor Manuel, Sikic Branimir I., Taylor Matthew Hiram, Pilja Lana, Hawthorne Thomas R., Yellin Michael Jay, Keler Tibor, Davis Thomas A. (2014). Phase I evaluation of an agonist anti-CD27 human antibody (CDX-1127) in patients with advanced hematologic malignancies. Journal of Clinical Oncology.

[105] Huang CT, Workman CJ, Flies D, Pan XY, Marson AL, Zhou G (2004). Role of LAG-3 in regulatory T cells. Immunity.

[106] Anderson AC, Joller N, Kuchroo VK (2016). Lag-3, Tim-3, and TIGIT: co-inhibitory receptors with specialized functions in immune regulation. Immunity.

[107] Gandhi MK, Lambley E, Duraiswamy J, Dua U, Smith C, Elliott S (2006). Expression of LAG-3 by tumor-infiltrating lymphocytes is coincident with the suppression of latent membrane antigen-specific CD8(+) T-cell function in Hodgkin lymphoma patients. Blood.

[108] Shapiro M, Herishanu Y, Ben Zion K, Dezorella N, Sun C, Kay S (2017). Lymphocyte activation gene 3: a novel therapeutic target in chronic lymphocytic leukemia. Haematologica.

[109] Wang J, Sanmamed MF, Datar I, Su TT, Ji L, Sun JW (2019). Fibrinogen-like protein 1 is a major immune inhibitory ligand of LAG-3. Cell.

[110] Kang XL, Kim J, Deng M, John S, Chen HY, Wu GJ (2016). Inhibitory leukocyte immunoglobulin-like receptors: Immune checkpoint proteins and tumor sustaining factors. Cell Cycle.

[111] Kang XL, Lu ZG, Cui CH, Deng M, Fan YQ, Dong BJ (2015). The ITIM-containing receptor LAIR1 is essential for acute myeloid leukaemia development. Nature Cell Biology.

[112] Zheng JK, Umikawa M, Cui CH, Li JY, Chen XL, Zhang CZ (2012). Inhibitory receptors bind ANGPTLs and support blood stem cells and leukaemia development. Nature.

[113] Deng M, Lu ZG, Zheng JK, Wan X, Chen XL, Hirayasu K (2014). A motif in LILRB2 critical for Angptl2 binding and activation. Blood.

[114] John S, Chen HY, Deng M, Gui X, Wu GJ, Chen WN (2018). A novel anti-LILRB4 CAR-T cell for the treatment of monocytic AML. Molecular Therapy.

[115] Aigner M, Feulner J, Schaffer S, Kischel R, Kufer P, Schneider K (2013). T lymphocytes can be effectively recruited for ex vivo and in vivo lysis of AML blasts by a novel CD33/CD3-bispecific BiTE antibody construct. Leukemia.

[116] Munoz L, Nomdedeu JF, Lopez O, Carnicer MJ, Bellido M, Aventin A (2001). Interleukin-3 receptor alpha chain (CD123) is widely expressed in hematologic malignancies. Haematologica.

[117] Jin LQ, Lee EM, Ramshaw HS, Busfield SJ, Peoppl AG, Wilkinson L (2009). Monoclonal antibody-mediated targeting of CD123, IL-3 receptor alpha chain, eliminates human acute myeloid leukemic stem cells. Cell Stem Cell.

[118] Kobold S, Pantelyushin S, Rataj F, vom Berg J (2018). Rationale for combining bispecific T cell activating antibodies with checkpoint blockade for cancer therapy. Frontiers in Oncology.

[119] Krupka C, Kufer P, Kischel R, Zugmaier G, Lichtenegger FS, Kohnke T (2016). Blockade of the PD-1/PD-L1 axis augments lysis of AML cells by the CD33/CD3 BiTE antibody construct AMG 330: reversing a T-cell-induced immune escape mechanism. Leukemia.

[120] Waldmann TA (2018). Cytokines in cancer immunotherapy. Cold Spring Harbor Perspectives in Biology.

[121] Charych DH, Hoch U, Langowski JL, Lee SR, Addepalli MK, Kirk PB (2016). NKTR-214, an engineered cytokine with biased IL2 receptor binding, increased tumor exposure, and marked efficacy in mouse tumor models. Clinical Cancer Research.

[122] Siefker-Radtke AO, Fishman MN, Balar AV, Grignani G, Diab A, Gao J (2019). NKTR-214+ nivolumab in first-line advanced/metastatic urothelial carcinoma (mUC): updated results from PIVOT-02. American Society of Clinical Oncology.

[123] Yu P, Steel JC, Zhang ML, Morris JC, Waldmann TA (2010). Simultaneous blockade of multiple immune system inhibitory checkpoints enhances antitumor activity mediated by interleukin-15 in a murine metastatic colon carcinoma model. Clinical Cancer Research.

[124] Garris CS, Arlauckas SP, Kohler RH, Trefny MP, Garren S, Piot C (2018). Successful anti-PD-1 cancer immunotherapy requires T cell-dendritic cell crosstalk involving the cytokines IFN-gamma and IL-12. Immunity.

[125] Quetglas JI, Labiano S, Aznar MA, Bolanos E, Azpilikueta A, Rodriguez I (2015). Virotherapy with a Semliki Forest virus-based vector encoding IL-12 synergizes with PD-1/PD-L1 blockade. Cancer Immunology Research.

[126] Guedan S, Posey AD, Shaw C, Wing A, Da T, Patel PR (2018). Enhancing CAR T cell persistence through ICOS and 4-1BB costimulation. JCI Insight.

[127] Chmielewski M, Abken H (2015). TRUCKs: the fourth generation of CARs. Expert Opinion on Biological Therapy.

[128] Locke FL, Ghobadi A, Jacobson CA, Miklos DB, Lekakis LJ, Oluwole OO (2019). Long-term safety and activity of axicabtagene ciloleucel in refractory large B-cell lymphoma (ZUMA-1): a single-arm, multicentre, phase 1-2 trial. The Lancet Oncology.

[129] Schuster SJ, Bishop MR, Tam CS, Waller EK, Borchmann P, McGuirk JP (2019). Tisagenlecleucel in adult relapsed or refractory diffuse large B-cell lymphoma. New England Journal of Medicine.

[130] Maude SL, Laetsch TW, Buechner J, Rives S, Boyer M, Bittencourt H (2018). Tisagenlecleucel in children and young adults with B-cell lymphoblastic leukemia. New England Journal of Medicine.

[131] D’Aloia MM, Zizzari IG, Sacchetti B, Pierelli L, Alimandi M (2018). CAR-T cells: the long and winding road to solid tumors. Cell Death & Disease.

[132] Sotillo E, Barrett DM, Black KL, Bagashev A, Oldridge D, Wu G (2015). Convergence of acquired mutations and alternative splicing of CD19 enables resistance to CART-19 immunotherapy. Cancer Discovery.

[133] Gardner R, Wu D, Cherian S, Fang M, Hanafi LA, Finney O (2016). Acquisition of a CD19-negative myeloid phenotype allows immune escape of MLL-rearranged B-ALL from CD19 CAR-T-cell therapy. Blood.

[134] Raponi S, De Propris MS, Intoppa S, Milani ML, Vitale A, Elia L (2011). Flow cytometric study of potential target antigens (CD19, CD20, CD22, CD33) for antibody-based immunotherapy in acute lymphoblastic leukemia: analysis of 552 cases. Leukemia & Lymphoma.

[135] Macauley MS, Crocker PR, Paulson JC (2014). Siglec-mediated regulation of immune cell function in disease. Nature Reviews Immunology.

[136] Fry TJ, Shah NN, Orentas RJ, Stetler-Stevenson M, Yuan CM, Ramakrishna S (2018). CD22-targeted CAR T cells induce remission in B-ALL that is naive or resistant to CD19-targeted CAR immunotherapy. Nature Medicine.

[137] Jen EY, Ko C-W, Lee JE, Del Valle PL, Aydanian A, Jewell C (2018). FDA Approval: gemtuzumab ozogamicin for the treatment of adults with newly diagnosed CD33-positive acute myeloid leukemia. Clinical cancer research.

[138] Sutherland MSK, Walter RB, Jeffrey SC, Burke PJ, Yu CP, Kostner H (2013). SGN-CD33A: a novel CD33-targeting antibody-drug conjugate using a pyrrolobenzodiazepine dimer is active in models of drug-resistant AML. Blood.

[139] Pizzitola I, Anjos-Afonso F, Rouault-Pierre K, Lassailly F, Tettamanti S, Spinelli O (2014). Chimeric antigen receptors against CD33/CD123 antigens efficiently target primary acute myeloid leukemia cells in vivo. Leukemia.

[140] Kenderian SS, Ruella M, Shestova O, Klichinsky M, Aikawa V, Morrissette JJD (2015). CD33-specific chimeric antigen receptor T cells exhibit potent preclinical activity against human acute myeloid leukemia. Leukemia.

[141] Wang QS, Wang Y, Lv HY, Han QW, Fan H, Guo B (2015). Treatment of CD33-directed chimeric antigen receptor-modified T cells in one patient with relapsed and refractory acute myeloid leukemia. Molecular Therapy.

[142] Kim MY, Yu KR, Kenderian SS, Ruella M, Chen S, Shin TH (2018). Genetic inactivation of CD33 in hematopoietic stem cells to enable CAR T cell immunotherapy for acute myeloid leukemia. Cell.

[143] Liu F (2018). First-in-human CLL1-CD33 compound CAR T cell therapy induces complete remission in patients with refractory acute myeloid leukemia: update on phase 1 clinical trial. Blood.

[144] Cai TY, Galetto R, Gouble A, Smith J, Cavazos A, Han LN (2017). Pre-clinical studies of allogeneic anti-CD123 CAR T-cells for the therapy of blastic plasmacytoid dendritic cell neoplasm (BPDCN). Blood.

[145] Al-Hussaini M, Rettig MP, Ritchey JK, Karpova D, Uy GL, Eissenberg LG (2016). Targeting CD123 in acute myeloid leukemia using a T-cell-directed dual-affinity retargeting platform. Blood.

[146] Mardiros A, Dos Santos C, McDonald T, Brown CE, Wang XL, Budde LE (2013). T cells expressing CD123-specific chimeric antigen receptors exhibit specific cytolytic effector functions and antitumor effects against human acute myeloid leukemia. Blood.

[147] Gill S, Tasian SK, Ruella M, Shestova O, Li Y, Porter DL (2014). Preclinical targeting of human acute myeloid leukemia and myeloablation using chimeric antigen receptor-modified T cells. Blood.

[148] Tasian SK, Kenderian SS, Shen F, Ruella M, Shestova O, Kozlowski M (2017). Optimized depletion of chimeric antigen receptor T cells in murine xenograft models of human acute myeloid leukemia. Blood.

[149] Zhang W, Stevens BM, Budde EE, Forman SJ, Jordan CT, Purev E (2017). Anti-CD123 CAR T-cell therapy for the treatment of myelodysplastic syndrome. Blood.

[150] Ruella M, Barrett DM, Kenderian SS, Shestova O, Hofmann TJ, Perazzelli J (2016). Dual CD19 and CD123 targeting prevents antigen-loss relapses after CD19-directed immunotherapies. Journal of Clinical Investigation.

[151] Tu S (2018). A novel chimeric antigen receptor T cells therapy strategy that dual targeting CD19 and CD123 to treat relapsed acute lymphoblastic leukemia after allogeneic hematopoietic stem cell transplantation. Blood.

[152] Luo Y, Chang L-J, Hu Y, Dong L, Wei G, Huang H (2015). First-in-man CD123-specific chimeric antigen receptor-modified T cells for the treatment of refractory acute myeloid leukemia. Blood.

[153] Tai YT, Anderson KC (2015). Targeting B-cell maturation antigen in multiple myeloma. Immunotherapy.

[154] Novak AJ, Darce JR, Arendt BK, Harder B, Henderson K, Kindsvogel W (2004). Expression of BCMA, TACI, and BAFF-R in multiple myeloma: a mechanism for growth and survival. Blood.

[155] Ali SA, Shi V, Maric I, Wang M, Stroncek DF, Rose JJ (2016). T cells expressing an anti-B-cell maturation antigen chimeric antigen receptor cause remissions of multiple myeloma. Blood.

[156] Brudno J, Lam N, Wang M, Stroncek D, Maric I, Stetler-Stevenson M (2017). T cells genetically modified to express an anti-B-cell maturation antigen chimeric antigen receptor with a CD28 costimulatory moiety cause remissions of poor-prognosis relapsed multiple myeloma. Blood.

[157] Cohen AD, Garfall AL, Stadtmauer EA, Lacey SF, Lancaster E, Vogl DT (2017). Safety and efficacy of B-cell maturation antigen (BCMA)-specific chimeric antigen receptor T cells (CART-BCMA) with cyclophosphamide conditioning for refractory multiple myeloma (MM). Blood.

[158] Zhao W-H (2018). Updated analysis of a phase 1, open-label study of LCAR-B38M, a chimeric antigen receptor T cell therapy directed against B-cell maturation antigen, in patients with relapsed/refractory multiple myeloma. Blood.

[159] Raje N, Berdeja J, Lin Y, Siegel D, Jagannath S, Madduri D (2019). Anti-BCMA CAR T-cell therapy bb2121 in relapsed or refractory multiple myeloma. New England Journal of Medicine.

[160] Mailankody S (2018). Clinical responses and pharmacokinetics of MCARH171, a human-derived Bcma targeted CAR T cell therapy in relapsed/refractory multiple myeloma: final results of a phase I clinical trial. Blood.

[161] van de Donk N, Richardson PG, Malavasi F (2018). CD38 antibodies in multiple myeloma: back to the future. Blood.

[162] Lin P, Owens R, Tricot G, Wilson CS (2004). Flow cytometric immunophenotypic analysis of 306 cases of multiple myeloma. American Journal of Clinical Pathology.

[163] Palumbo A, Chanan-Khan A, Weisel K, Nooka AK, Masszi T, Beksac M (2016). Daratumumab, bortezomib, and dexamethasone for multiple myeloma. New England Journal of Medicine.

[164] Feng XY, Zhang L, Acharya C, An G, Wen K, Qiu LG (2017). Targeting CD38 suppresses induction and function of T regulatory cells to mitigate immunosuppression in multiple myeloma. Clinical cancer research.

[165] Mihara K, Yanagihara K, Takigahira M, Kitanaka A, Imai C, Bhattacharyya J (2010). Synergistic and persistent effect of T-cell immunotherapy with anti-CD19 or anti-CD38 chimeric receptor in conjunction with rituximab on B-cell non-Hodgkin lymphoma. British Journal of Haematology.

[166] Drent E, Themeli M, Poels R, de Jong-Korlaar R, Yuan HP, de Bruijn J (2017). A rational strategy for reducing on-target off-tumor effects of CD38-chimeric antigen receptors by affinity optimization. Molecular Therapy.

[167] O'Connell FP, Pinkus JL, Pinkus GS (2004). CD138 (Syndecan-1), a plasma cell marker - immunohistochemical profile in hematopoietic and nonhematopoietic neoplasms. American Journal of Clinical Pathology.

[168] Heffner LT, Jagannath S, Zimmerman TM, Lee KP, Rosenblatt J, Lonial S (2012). BT062, an antibody-drug conjugate directed against CD138, given weekly for 3 weeks in each 4 week cycle: safety and further evidence of clinical activity. Blood.

[169] Guo B, Chen MX, Han QW, Hui F, Dai HR, Zhang WY (2016). CD138-directed adoptive immunotherapy of chimeric antigen receptor (CAR)-modified T cells for multiple myeloma. Journal of Cellular Immunotherapy.

[170] Rafiq S, Yeku OO, Jackson HJ, Purdon TJ, van Leeuwen DG, Drakes DJ (2018). Targeted delivery of a PD-1-blocking scFv by CAR-T cells enhances anti-tumor efficacy in vivo. Nature Biotechnology.

[171] Fraietta JA, Lacey SF, Orlando EJ, Pruteanu-Malinici I, Gohil M, Lundh S (2018). Determinants of response and resistance to CD19 chimeric antigen receptor (CAR) T cell therapy of chronic lymphocytic leukemia. Nature Medicine.

[172] Galon J, Rossi J, Turcan S, Danan C, Locke FL, Neelapu SS (2017). Characterization of anti-CD19 chimeric antigen receptor (CAR) T cell-mediated tumor microenvironment immune gene profile in a multicenter trial (ZUMA-1) with axicabtagene ciloleucel (axi-cel, KTE-C19). Journal of Clinical Oncology.

[173] Zolov SN, Rietberg SP, Bonifant CL (2018). Programmed cell death protein 1 activation preferentially inhibits CD28.CAR-T cells. Cytotherapy.

[174] Hui EF, Cheung J, Zhu J, Su XL, Taylor MJ, Wallweber HA (2017). T cell costimulatory receptor CD28 is a primary target for PD-1-mediated inhibition. Science.

[175] Liu XJ, Zhang YP, Cheng C, Cheng AW, Zhang XY, Li N (2017). CRISPR-Cas9-mediated multiplex gene editing in CAR-T cells. Cell Research.

[176] Li A (2018). Checkpoint inhibitors augment CD19-directed chimeric antigen receptor (CAR) T cell therapy in relapsed B-cell acute lymphoblastic leukemia. Blood.

